# Phenotypic Variation of *Botrytis cinerea* Isolates Is Influenced by Spectral Light Quality

**DOI:** 10.3389/fpls.2020.01233

**Published:** 2020-08-13

**Authors:** Lijuan Meng, Hanna Mestdagh, Maarten Ameye, Kris Audenaert, Monica Höfte, Marie-Christine Van Labeke

**Affiliations:** Department of Plants and Crops, Faculty of Bioscience Engineering, Ghent University, Ghent, Belgium

**Keywords:** gray mold, phenotypical variability, pathogenicity, strawberry, red light, image-based early detection

## Abstract

*Botrytis cinerea*, a fungal pathogen that causes gray mold, displays a high degree of phenotypic diversity. Light emitting diodes (LEDs) with specific light spectrum are increasingly used as lighting resource for plant greenhouse production. The chosen light spectrum can also have an effect on the pathogens in this production system. In this study, we investigated the phenological diversity in 15 *B. cinerea* isolates upon different light treatments. Daylight, darkness, and LED lights with different wavelengths (white, blue, red, blue+red) were chosen as treatments. The 15 *Botrytis* isolates differed in their mycelial growth rate, conidia production, and sclerotia formation. Light quality had a limited effect on growth rate. All isolates sporulated under daylight treatment, red light resulted in lower sporulation, while white, blue, and blue+red light inhibited sclerotia formation in all isolates, and sporulation in most, but not all isolates. Pathogenicity of the *Botrytis* isolates was studied on 2-week-old strawberry (*Fragaria × ananassa* ‘Elsanta’) leaves grown under white, blue, and red LED lights. The isolates differed in virulence on strawberry leaves, and this was positively correlated to oxalic acid production by *B. cinerea in vitro*. Red LED light improved leaf basal resistance to all the tested *Botrytis* isolates. Blue light pretreatment resulted in decreased leaf resistance to some isolates. Furthermore, we used image analysis to quantify the virulence of the different *Botrytis* isolates based on changes in photosynthetic performance of the strawberry leaves: chlorophyll fluorescence (F_v_/F_m_), chlorophyll index (ChlIdx) and anthocyanin content (modified anthocyanin reflection index, mAriIdx). F_v_/F_m_ showed a strong negative correlation with disease severity and can be an indicator for the early detection of gray mold on strawberry leaves.

## Introduction

*Botrytis cinerea* Pers.:Fr the causal agent of gray mold disease is a filamentous, heterothallic fungus with a necrotrophic life style (Alfonso et al., 2000). This pathogen has a wide host range and infects more than 1,000 plant species worldwide including vegetables, ornamentals, and fruits, leading to important yield and quality losses (Elad et al., 2016). *B. cinerea* is a highly versatile pathogen. As a necrotroph, it can extract nutrients from dead or senescent plant material, but it can also infect living tissues *via* direct penetration or through natural openings or wounds (Williamson et al., 2007). Additionally, it can also grow saprophytically. *Botrytis* can be isolated from different plant species in nature and infection can be reproduced in the laboratory on a wide range of hosts. Yet, a certain degree of host specialization exists in this pathogen (Thompson and Latorre, 1999; Muñoz et al., 2002). For example, Cotoras and Silva (2005) reported that *B. cinerea* strains isolated from tomato were more virulent on tomato leaves than isolates from grapes. Furthermore, a study of 490 isolates from open-field crops by microsatellite loci suggested the occurrence of host-specific divergence of *B. cinerea* in perennial hosts (Asadollahi et al., 2013).

*B. cinerea* isolates show phenotypic and genetic variability. Differences in colony morphology, mycelial growth, sporulation intensity, sclerotia formation, and pathogenicity have been described (Di Lenna et al., 1981; Martinez et al., 2003; Khazaeli et al., 2010; Pande et al., 2010; Kuzmanovska et al., 2012; Kumari et al., 2014). *B. cinerea* produces a battery of extracellular enzymes, including pectinases and pectin methylesterases (Reignault et al., 1994). *Botrytis* isolates with different pathogenic capabilities on various host produced different amounts of extracellular pectic enzymes (Di Lenna et al., 1981). High genetic diversity in *B. cinerea* populations has been revealed using a multiplicity of molecular techniques, such as PCR detection of transposable elements (Martinez et al., 2008), restriction fragment length polymorphism (RFLP) analysis of PCR-amplified loci (Baraldi et al., 2002; Muñoz et al., 2002), PCR amplification of microsatellite loci (Isenegger et al., 2008; Asadollahi et al., 2013), and randomly amplified polymorphic DNA (RAPD) analysis (Alfonso et al., 2000; Pande et al., 2010). Disease control is difficult because the pathogen has a broad host range and it can survive as mycelium and/or conidia or as sclerotia for extended periods. In addition, *B. cinerea* isolates differ in their sensitivity to fungicides and fungicide resistance is quickly obtained (Kretschmer and Hahn, 2008).

*B. cinerea* has 11 photoreceptors including 2 cryptochromes, 4 LOVs (light, oxygen, voltage), 2 opsins, and 3 phytochromes to respond to different light conditions varying from near-UV, blue, green, red, and far-red light (Schumacher, 2017). Blue light is sensed by the proteins that bind flavin *via* LOV or FAD (flavin adenine dinucleotide) domains such as BcWCL1 and BcWCL2 (the orthologs of white collar complex in *B. cinerea*), and BcVVD1 (the orthologs of vivid in *B. cinerea*) (Rodriguez-Romero et al., 2010). BcWCL1 interacts with BcWCL2 in the nuclei, forming the white collar complex (WCC), which is required to respond to white light (Schumacher, 2012). Opsins are transmembrane proteins using retinal to sense green light, and phytochromes are histidine kinases using bilin to perceive red/far-red light (Rodriguez-Romero et al., 2010). Based on the light perception by these photoreceptors, *B. cinerea* senses surrounding light as a decision-tool for morphogenesis, as a guide for directed growth, as a stress factor for protection, and also as a time giver for the circadian clock (Schumacher, 2017). Studies have described the impact of light on mycelial growth, conidiation, sclerotial development, and tropic response, however, unclear results have been reported caused by *B. cinerea* isolate variability and different experimental conditions (Schumacher, 2017).

Strawberry (*Fragaria × ananassa*) is an important soft fruit crop that is popular all over the world. Gray mold is a serious disease in strawberry production and leads to important economic losses (Debode et al., 2015; Petrasch et al., 2019). *B. cinerea* can infect all plant parts of strawberry including leaves, fruits, flowers, petioles, and stems at every growth stage (Williamson et al., 2007; Petrasch et al., 2019). In year-round greenhouse strawberry production, light emitting diodes (LED) are increasingly applied to increase the day length as well as the light intensity in winter. LED lighting offers the possibility of spectral modulation thus influencing both morphology and metabolite content of the leaves. Commercial LED lamps typically combine blue and red wavelengths as these are highly absorbed by chlorophyll and thus promote photosynthesis and biomass production (Okamoto et al., 1996). Application of LED lighting also opens the possibility to increase the strawberry’s resistance to *B. cinerea.* Indeed, we previously showed that leaves that develop under monochromatic red light are more resistant to *Botrytis* infection compared to white, blue, and blue+red lights (Meng et al., 2019). Yet, in aforementioned study only one *Botrytis* strain was investigated. Hence, the investigation of the diversity in light-response of different *B. cinerea* isolates is of importance for the LED light application in greenhouse production.

Given the facts described above, we hypothesized that *B. cinerea* isolates will differentially respond to different light qualities. Therefore, we investigated the light-modulated phenotypic diversity of 15 *B. cinerea* isolates. We characterized their pathogenicity on strawberry leaves under white, blue, and red LED lights and hypothesized that a pretreatment of leaves with red light would reduce disease susceptibility irrespective of the isolate. To assess the virulence of the different *Botrytis* isolates on strawberry leaves in an objective way, we investigated the potential of image-based early detection to quantify changes in the photosynthetic performance of the leaves (quantum efficiency of photosystem II, chlorophyll index) and/or leaf defense compounds (modified anthocyanin reflection index).

## Materials and Methods

### *Botrytis cinerea* Isolates

Fifteen *B. cinerea* isolates were used in this study (**Table 1**). Four *B. cinerea* isolates (B1, B2, B6, and B7) are well-documented laboratory strains. Eleven gray mold isolates were collected from tomato, lettuce, apple, and grape in Belgium. Purified isolates were cultivated on potato dextrose agar (PDA, Becton, Dickinson, and Company) and subjected to long-term storage in 20% glycerol at −80°C.

**Table 1:** *Botrytis cinerea* isolates used in this study.

*Botrytis* isolates*	Host plant	Origin/geographic origin	References
B1 (R16)	Grape	Result of the crossing SAS56×SAS405	(Faretra and Pollastro, 1991)
B2 (Bd90)	Grape	Bordeaux (France)	(Reignault et al., 1994)
B3	Lettuce	Belgium, 2018	this study
B4	Tomato	Belgium, 2018	this study
B5	Lettuce	Belgium, 2018	this study
B6 (B05.10)	Grape	Haploid strain resulted from a treatment with benomyl in Germany	(Quidde et al., 1998)
B7 (A336)	Grape	mutant of Bd90, from Bordeaux (France)	(Hamada et al., 1994)
B8	Lettuce	Belgium, 2018	this study
B9	Lettuce	Belgium, 2018	this study
B10	Apple	Belgium, 2018	this study
B11	Grape	Belgium, 2018	this study
B12	Lettuce	Belgium, 2018	this study
B13	Tomato	Belgium, 2018	this study
B14	Lettuce	Belgium, 2018	this study
B15	Lettuce	Belgium, 2018	this study

*Alternative names are indicated between brackets.

### Light Quality Treatments to Study Botrytis Phenotypes

To investigate mycelial growth, sporulation, and sclerotia production, the 15 *B. cinerea* isolates were grown on PDA under different spectral light qualities at 20°C. Six different light regimes were established using 1) daylight, 2) white LEDs (300–800 nm, 29% B, 39% G, 27% R, and 5% FR, Philips, the Netherlands), 3) blue LEDs (400–500 nm, peak at 460 nm), 4) red LEDs (600–700 nm, peak at 660 nm), 5) red + blue LEDs (75/25%, peak at 660 nm and 460 nm) (**Supplementary Figure 1**) and (6) dark as control. A photoperiod of 16 h and a photon flux density of 40 µmol m^−2^ s^−1^ were provided by the LEDs. Natural daylight was provided at a lab bench (18°C), with a natural day length of 15 h and average photosynthetic photon flux density (PPFD) of 10 µmol m^−2^ s^−1^. The spectral light distribution and the light intensity were measured using a spectroradiometer (JAZ-ULM-200, Ocean Optics, US).

Mycelial plugs with 6 mm in diameter were inoculated in the center of Petri dishes (90 mm in diameter). The Petri dishes were assigned to one of the six light treatments. Six replicates per isolate and per light treatment were used. The radial growth per colony was measured daily on two perpendicular axes for 5 days or until it reached the edge of the plate. The growth rate (cm day^−1^) was calculated as the average growth length increase per day.

To assess the sporulation and sclerotia production, PDA plates inoculated with 6 mm-mycelial plugs remained under the six light treatments for 15 days. Sporulation and sclerotia production were assessed visually and by microscopy. The class system was set as: class 0 (no spores/sclerotia formation); class I; (very few spores/sclerotia formation); class II (sparse sporulation/sclerotia formation); class III (average amount spores/sclerotia formation); class IV (many spores/sclerotia formation); class V (abundant formation of spores/sclerotia). The classification system for sclerotia is illustrated in **Supplementary Figure 2**. This was repeated three times with six replicates each time (n = 18).

### Oxalic Acid Detection Assay

Plugs of *B. cinerea* isolates were inoculated on complete medium (Canessa et al., 2013) and always maintained in the dark at 24°C. This pH-indicating medium contains 0.1% bromothymol blue as indicator, and the medium color changes from green to yellow when an acid compound such as oxalic acid is produced. Medium acidification was evaluated after 7 days by its effect on the medium pH. The pH was measured on the outside of the yellow circle using a flat pH electrode (SF113, VWR, Germany) which can test the pH directly *via* the surface of the medium. Four measurements were conducted for each plate and averaged, this was done in four replications per isolate.

### Pathogenicity Test on Strawberry

Strawberry (*Fragaria* × *ananassa* ‘Elsanta’) leaves that developed under different light qualities were used for the pathogenicity tests with the 15 *B.* *cinerea* isolates. The plants were potted in peat substrate (Van Israel nv, Belgium) and raised in a growth chamber with 70% relative humidity at 20°C. The growth chamber was equipped with three light qualities: white (W, 300–800 nm, Philips, the Netherlands), blue (B, peak at 460 nm, Philips, the Netherlands), and red (R, peak at 660 nm, Philips, the Netherlands) LED lights (**Supplementary Figure 1**). A photoperiod of 16 h with the photosynthetic photon flux density at 100 µmol m^−2^ s^−1^ was provided. The plants were fertilized with Soluplant (N:P:K:Ca 19-8-16-4, Haifa, the Netherlands, EC=1.5 dS/m, pH=5.7) three times per week.

All the 15 isolates were tested on strawberry leaves grown under white LED light, and a subset of 10 isolates was tested on leaves that developed under blue and red LED lights. *Botrytis* isolates were cultivated on PDA in Petri dishes. After 7–10 days, conidia were washed from the plates with ¼ potato dextrose broth (PDB) solution containing 0.01% (v/v) Tween 20. After removing the mycelium fragments, spore titers were determined microscopically using a Thoma counting chamber. A final concentration of 5×10^5^ spore ml^−1^ was used for inoculation. Leaf discs of 1-cm in diameter from 2-week-old strawberry leaves were cut the day before inoculation and placed in disposable 24-well plates with water. Each leaf disc was inoculated with 10 µl droplets of conidial suspension on the adaxial leaf surface. Incubation was at 22°C under dark conditions. Disease symptoms were scored after 3 days. A 0–3 ordinal rating scale was employed for disease rating and disease index was calculated. Six leaf discs per leaf with four biological replicates were used in this study.

### Image Analysis

Non-invasive spectral phenotyping was applied to monitor the strawberry disease development by a platform that allows to visualize diverse physiological traits in real time, based on specific absorption, reflection, and fluorescence patterns in visible and near-infrared (NIR) wavelengths. The central part of the platform comprises a 3CCD 6 Mp—16 bit camera mounted on a Cartesian coordinate robot, equipped with 12 optical interference filters (CropReporter, PhenoVation B.V., Wageningen, the Netherlands).

This multispectral imaging platform was used daily to record the lesion development of inoculated leaf discs floating in 24-well plates (24 replicates per light quality treatment and *Botrytis* isolate) from the day of inoculation until 4 days post-inoculation (dpi).

RGB (red green blue) images, reflectance spectra to calculate the anthocyanin index and chlorophyll index and the minimal fluorescence, F_0_, and the maximum fluorescence, F_m_, are captured by the camera. Images obtained from the phenotyping platform were processed *via* the “Data Analysis Software” program (PhenoVation B.V., Wageningen, the Netherlands).

The modified anthocyanin reflectance index (mAriIdx) was determined using following formula (Gitelson et al., 2009):

$$\text{mAriIdx}=\left(\frac{1}{\rho_{550\text{nm}}}-\frac{1}{\rho_{710\text{nm}}}\right)\rho_{770\text{nm}}$$

The chlorophyll index (ChlIdx) was calculated using following formula (Gitelson et al., 2009):

$$\text{ChlIdx}=\left(\frac{\rho_{770\text{nm}}}{\rho_{710\text{nm}}}-1\right)$$

where ρ_550_ is the reflectance in the first spectral band, which is maximally sensitive to anthocyanin content; ρ_710_ the reflectance in the second spectral band, which is maximally sensitive to chlorophyll content but not sensitive to anthocyanin content; and ρ_770_ the reflectance of the third spectral band, which compensates for leaf thickness and density.

The maximum quantum efficiency of photosystem II (F_v_/F_m_) was calculated using following formula (Baker, 2008):

$$F_{v}/F_{m}=(F_{m}-F_{0})/F_{m}$$

Between measurements, the 24-well plates were placed in the dark allowing immediate quantification of the minimal (F_0_), then saturating red light flashes of 3,000 µmol m^−2^ s^−1^ were given. This allows the imaging of the OJIP induction curve at 12 images per second at a resolution of 1.5 Mp. This resolution gives the optimal signal to noise with respect to the detail in the image (PhenoVation B.V., Wageningen, the Netherlands). From the F_0_ and F_m_ image measurements based on the OJIP induction curve the variable fluorescence F_v_/F_m_ is calculated (Björkman and Demmig, 1987; Kalaji et al., 2014). This yields images of F_v_/F_m_ and from these images corresponding average values including standard deviation of the whole image are calculated (imaging software and algorithms by PhenoVation B.V., Wageningen, the Netherlands).

### Statistical Analysis

Data were tested for normal distribution using the Kolmogorov–Smirnov test and for homoscedasticity of variances using Levene’s test. The mycelial growth rate and disease rating among *B. cinerea* isolates were compared by the non-parametric Kruskal-Wallis test with Dunn test as the *post hoc* test. The effect of light quality on mycelial growth rate was analyzed by one-way ANOVA. If significant differences were found, the Tukey test (p ≤ 0.05) was carried out to establish significant differences between means, here a Bonferroni correction was applied when n ≥ 10. The virulence of the *Botrytis* isolates assessed by the multispectral camera (F_v_/F_m_, ChlIdx, mAriIdx) were analyzed by one-way ANOVA for each light quality. As the light pretreatment affects both chlorophyll and polyphenol content (Meng et al., 2019), the effects of the light quality pretreatments were analyzed by ANCOVA using the images at the start (day 0) as covariate, adjusted means were calculated using Bonferroni for confidence interval adjustment. All assumptions for performing ANCOVA including homogeneity of regression slopes were checked. All analyses were performed using SPSS version 26 (SPSS Inc., Chicago, USA).

## Results

### Phenotypic Characterization of *B. cinerea* Isolates

**Figure 1:**
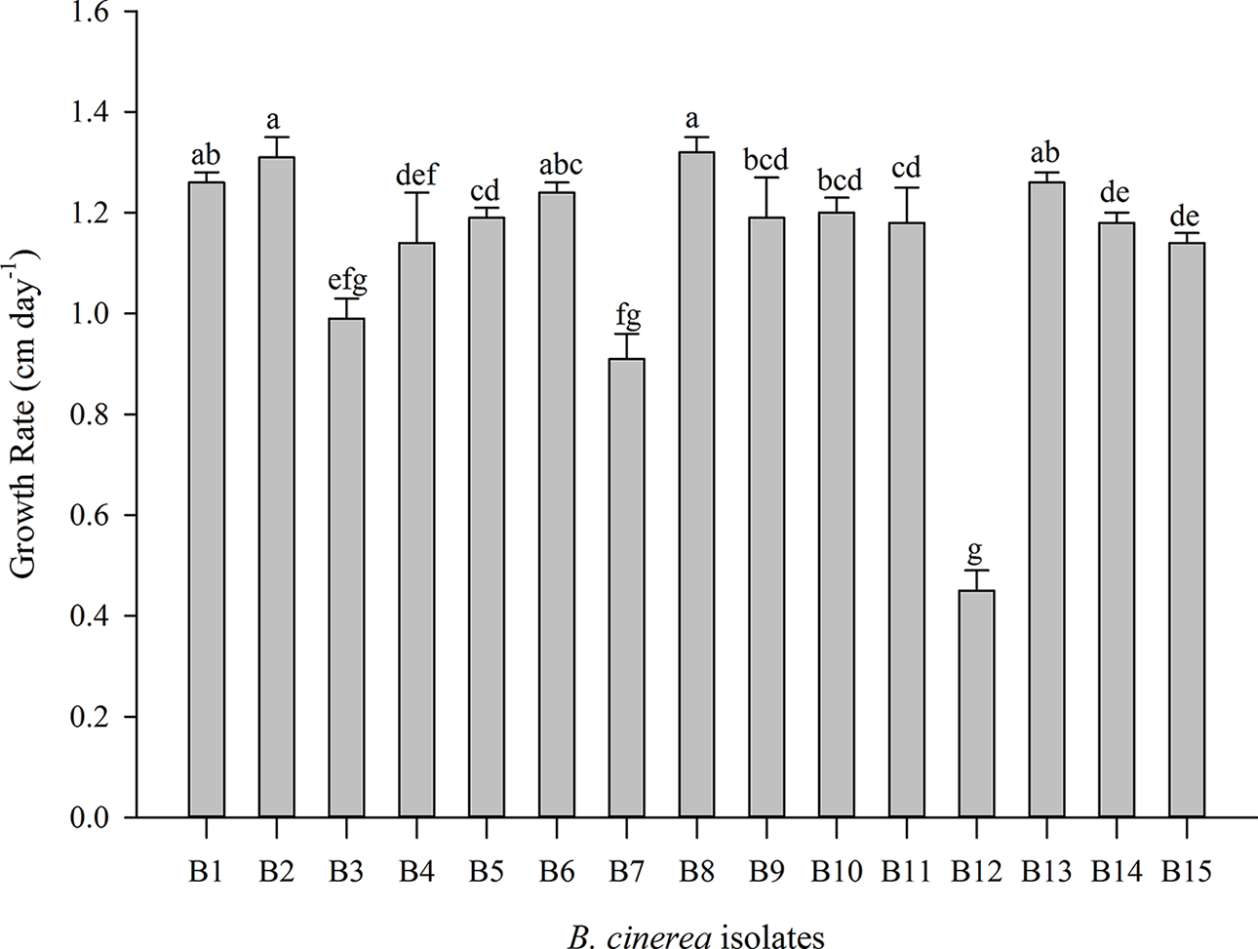
Mycelial growth rate of 15 *Botrytis cinerea* isolates in dark condition. Data are presented as mean of six replicates with standard deviation. Different letters indicate statistical differences among *B. cinerea* isolates based on Kruskal-Wallis test with Bonferroni correction (Dunn test, p ≤ 0.0033).

Significant differences in the mycelial growth rate were observed between the 15 *B. cinerea* isolates, this for the dark control treatment (**Figure 1**, **Supplementary Table 1**). B12 resulted in the lowest growth rate (0.45 cm day^−1^) while B2 and B8 had a ± three-fold higher growth rate (respectively 1.31 and 1.32 cm day^−1^).

**Figure 2:**
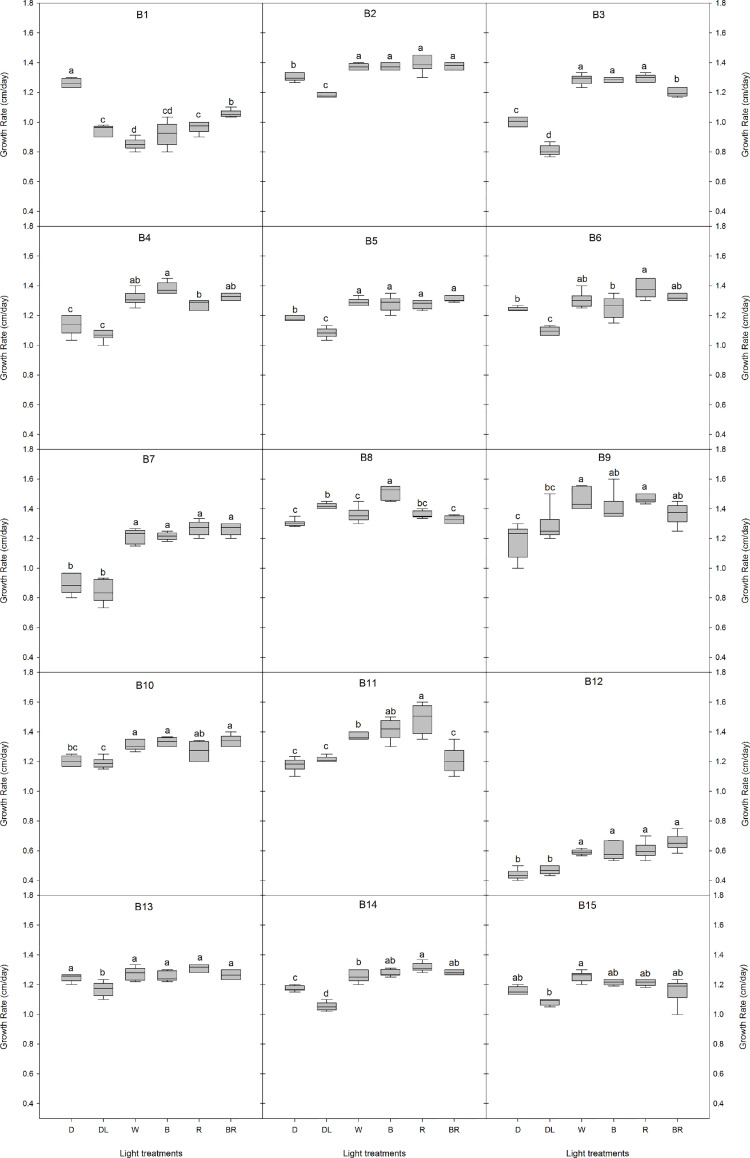
Effects of light quality on the growth rate of 15 *Botrytis cinerea* isolates. Six light treatments were analyzed namely dark (D), daylight (DL), white (W), blue (B), red (R), and blue+red (BR). Different letters indicate statistical differences between light treatments for each isolate based on Tukey’s test (p ≤ 0.05), except for B2 and B15 where a non-parametric Kruskal-Wallis followed by a *post-hoc* Dunn’s test (p ≤ 0.05) was performed.

Light conditions greatly influenced the growth rate of *B. cinerea* (**Figure 2**, **Supplementary Table 1**). For the isolates B2, B3, B4, B5, B7, B12, and B14, the mycelial growth was considerable higher under LED lights (white, blue, red, blue+red) compared to the dark and daylight treatment. The spectral quality of the LED treatment did not affect the growth rate of B2, B5, B7, B9, B10, B12, B13, and B15. However, compared to white LED light, a significant increase was observed in the growth rate of B8 under blue light, while red light enhanced the growth rate of B1, B11, and B14. The combination blue+red, decreased the mycelial growth of B3 and B11 considerably, while an increase was observed in B1. Additionally, B1 was the only isolate where the highest growth rate was observed in the dark while all other isolates had higher or equal growth rates than the dark treatment.

**Figure 3:**
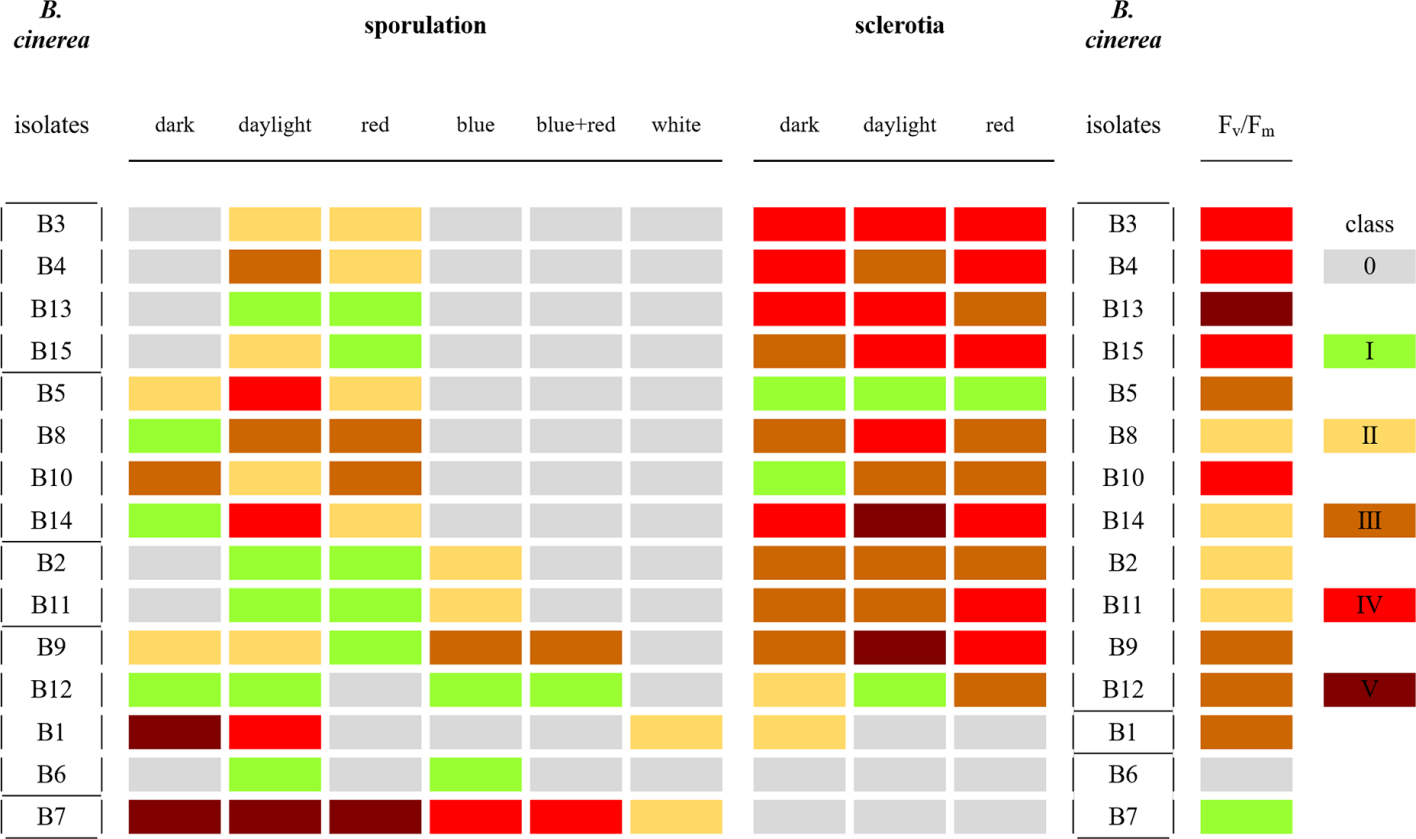
Classification of sporulation and sclerotia formation in the 15 *Botrytis cinerea* isolates after 15 days under six light treatments: dark, daylight, white, blue, red, and blue+red LED, and classification based on F_v_/F_m_ values at 4 dpi from infection in white-light-leaves. Classes of sporulation and sclerotia presented are from three replications with six plates for each replicate (n = 18). The classes of sporulation and sclerotia formation are: class 0 (no spores/sclerotia formation); class I (very few spores/sclerotia formation); class II (sporulation/sclerotia formation sparse); class III (average amount of spores/sclerotia formation); class IV (many spores/sclerotia formation); class V (abundant spore/sclerotia formation). The class of virulence is based on the significant letters of F_v_/F_m_ at 4 dpi (**Table 2**) with exclusion of B6 which is indicated by a light gray color. The classes are: class I (significant letters starting with a); class II (significant letters starting with b); class III (significant letters starting with c); class IV (significant letters starting with d); class V (significant letters starting with e). These classes are shown in colors (see the color bar).

Sporulation and sclerotia formation varied among the 15 *B. cinerea* isolates, and both were considerably affected by the light treatments used in this study (**Figure 3**). The 15 isolates were grouped according to the presence/absence of sporulation and of sclerotia formation under the light treatments (**Figure 3**). Four groups were defined based on their sporulation response. A first group clusters two lettuce and two tomato isolates (B3, B4, B13, B15) as they only produced spores under daylight and red LED light. A second group, including three lettuce and one grape isolate (B5, B8, B10, B14) sporulated in dark condition as well as under daylight and red light. The third group (grape isolates B2 and B11) developed spores under daylight, red and blue light. The fourth group, including grape and lettuce isolates (B1, B6, B9, and B12) exhibited great variability in sporulation compared to the other isolates under the considered light conditions. Finally, the nonpathogenic mutant B7 (A366) forms a fifth group and produced spores in all six treatments.

No sclerotia formation was observed under white, blue, and blue+red LED lights, this for all isolates. Three groups could be distinguished based on the formation of sclerotia in dark, under daylight or under red light (**Figure 3**). No sclerotia were produced in B6 (B05.10) and B7 (A366) irrespectively the light or dark treatment, resulting in a first group. B1 (R16) formed a second group with only sclerotia formation in the dark. The third group is formed by the remaining twelve isolates including lettuce, tomato, apple, and grape isolates, as they all produced sclerotia under dark, daylight, and red LEDs.

**Figure 4:**
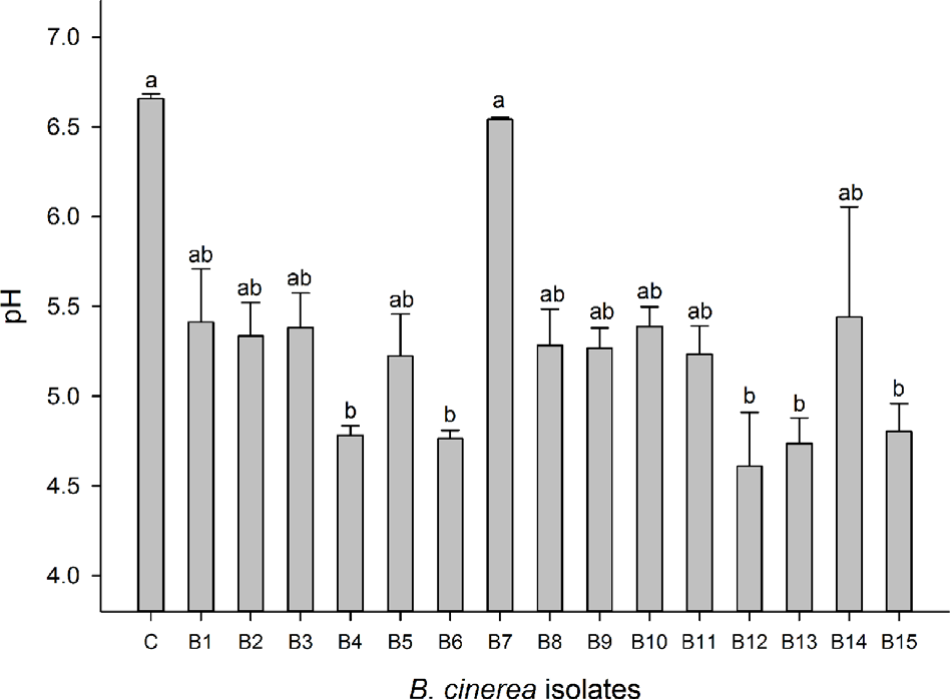
Oxalic acid production of *Botrytis cinerea* isolates was assessed based on changes in the medium pH. Control treatment (C) is medium without *Botrytis* inoculation. Data are shown as means ± SE (n=4). Different letters indicate statistical differences between *B. cinerea* isolates. Non-parametric Kruskal-Wallis followed by a *post-hoc* Dunn’s test was performed with p ≤ 0.05.

Oxalic acid formation can be indicative as a virulence factor for *Botrytis* (Williamson et al., 2007; Sun et al., 2019). Media acidification due to organic acid formation was strongest for B4, B6, B12, B13, and B15, resulting in a decrease of more than 1.5 pH units compared to a non-inoculated control. The nonpathogenic mutant B7 (A366) did not acidify the medium in comparison to the control. All other isolates caused an intermediate acidification reducing the medium with 1.0 pH unit in comparison to the control (**Figure 4**). White, blue, and red LED irradiation had no significant effect on media acidification caused by different *Botrytis* isolates (data are not shown).

### Inoculation Assays on Strawberry Leaves

**Figure 5:**
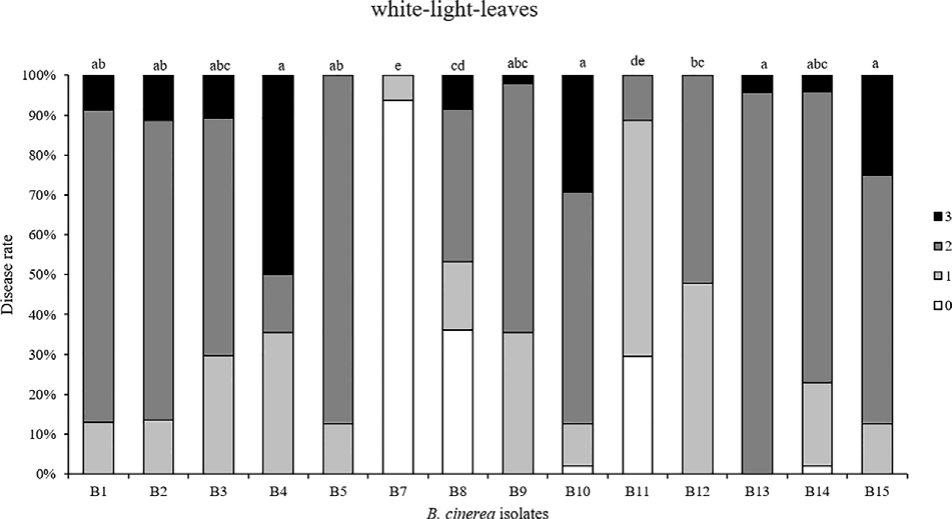
Disease severity caused by 14 *Botrytis cinerea* isolates spore-inoculated on strawberry leaves grown under white LED lights. Disease rating was scored 3 days post-inoculation using four scoring categories (0, resistant; 1, slightly spreading lesion; 2, moderately spreading lesion; 3, severely spreading lesion). B6 was not tested because not enough spores were produced. Different letters indicate statistical differences among the isolates performed by Kruskall-Wallis test followed by a *post-hoc* Dunn’s test with Bonferroni correction (p ≤ 0.0036, n = 4).

First, 14 *Botrytis* isolates were tested by spore inoculation on white LED light-developed strawberry leaves. B6 could not be included because of its poor sporulation (**Figure 5**). Different degrees of virulence on strawberry leaves were observed between these 14 isolates. B4, B10, B13, and B15 were the most virulent, followed by B1, B2, B3, B5, B9, and B14. An intermediate virulence was observed for B8, B11, and B12. As expected, strawberry leaves were hardly infected by the non-pathogenic B7 (A366) strain.

**Figure 6:**
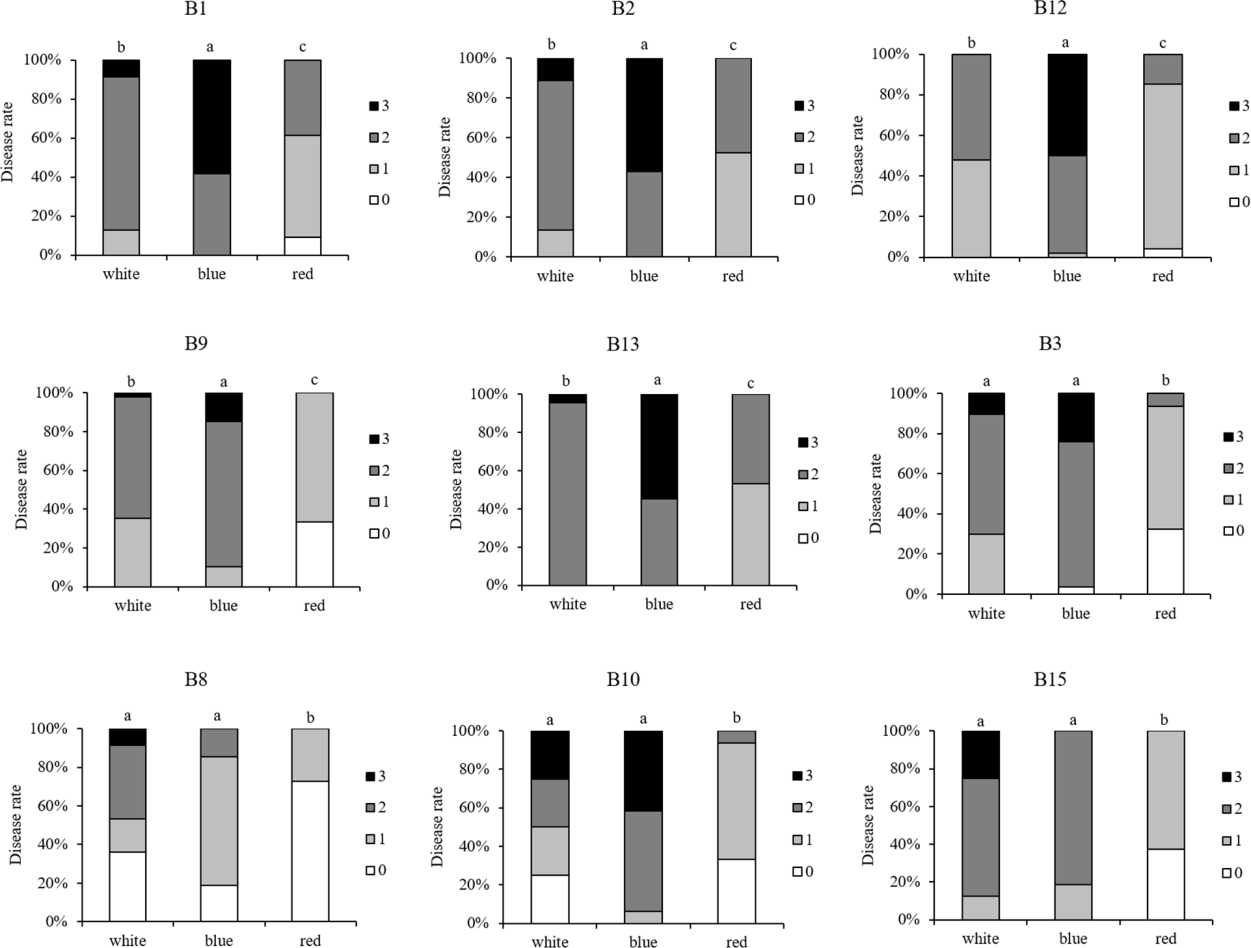
Comparison of virulence per isolate (including B1, B2, B3, B8, B9, B10, B12, B13, B15) with respect to the light pretreatments of the strawberry leaves. For each *Botrytis* isolate, virulence was tested on strawberry leaves grown under white, blue, and red LED lights, spore-inoculated leaf discs were dark incubated after inoculation and disease severity was compared between white-, blue-, and red-light-developed leaves. Disease rating was scored 3 days post-inoculation using four scoring categories (0, resistant; 1, slightly spreading lesion; 2, moderately spreading lesion; 3, severely spreading lesion). Different letters indicate statistical differences among the isolates performed by Kruskall-Wallis followed by a *post-hoc* Dunn’s test (p ≤ 0.05).

Second, ten *Botrytis* isolates were tested on strawberry leaves that were developed under blue and red LED lights. The isolates showed significant variations in their virulence (**Supplementary Figures 3A, B**). B7 (A366) was again the least aggressive, while B1, B2, and B13 were the most aggressive isolates and B15 resulted in moderate disease rating on the leaves that had developed either under blue or red light. Strawberry leaves that originated from red light were more resistant against the tested *Botrytis* isolates compared to leaves from white or blue light (**Figure 6**). Blue-light-developed leaves were more susceptibility to B1, B2, B9, B12, B13, while no remarkable difference was noted for isolates B3, B8, B10, B15, when compared to white-light-developed leaves. The isolate B7 (A366) also remained non-virulent on blue or red light-developed leaves (**Supplementary Figures 3A, B**).

Overall, B8 (lettuce isolate) was the least aggressive *Botrytis* isolate on strawberry leaves irrespective of light treatments, while B13 (tomato isolate) was the most aggressive one. Interestingly, B15 (lettuce isolate) resulted in the strongest disease symptoms on leaves from white light, however, blue- and red-light developed leaves displayed moderate resistance to B15.

### Image-Based Assessment of Botrytis Infection on Strawberry Leaves

**Figure 7:**
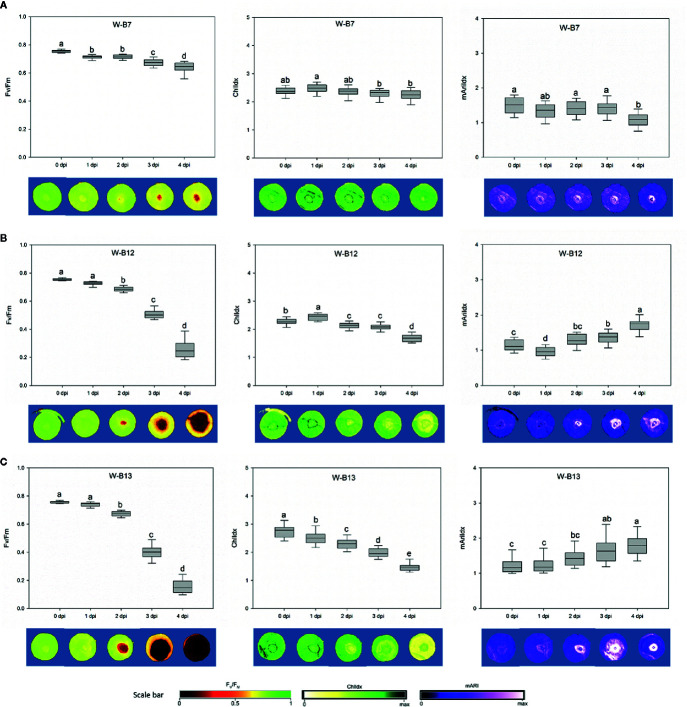
The variations of F_v_/F_m_, ChlIdx, and mAriIdx from 0 to 4 dpi caused by *Botrytis cinerea* isolates B7 **(A)**, B12 **(B)**, and B13 **(C)** were correlated with the development of disease lesion on white-light-leaves. The corresponding images are presented underneath the figures. Disease lesion led to darker F_v_/F_m_ image with lower value, yellower ChlIdx image with lower value, and brighter mAriIdx image with higher level. One-way ANOVA was applied for the statistical analysis (Tukey test, p ≤ 0.05), and data are shown by box plots with median. Different letters indicates significant differences among the time points.

For the image-based assessments of the progress rate of the disease after spore inoculation, changes in both chlorophyll fluorescence imaging (F_v_/F_m_) and stress indices (ChlIdx and mAriIdx) were assessed during 5 days (**Figure 7**, **Supplementary Figures 4**-**6**). The phenotyping of the plant resistance is shown from 0 to 4 dpi for three representative *Botrytis* isolates: the non-pathogenic B7 strain, the intermediate aggressive B12, and the most aggressive B13 isolate as determined by visual scoring on white-light-developed leaves (**Figure 5**). The non-pathogenic B7 displayed a minor but significant decrease in F_v_/F_m_ from 0.755 at 0 dpi to 0.637 at 4 dpi and hardly any change in ChlIdx and mAriIdx (**Figure 7A**). For B12 a strong decline was observed in F_v_/F_m_ from 3 dpi on, with values decreasing from 0.755 at 0 dpi to 0.263 at 4 dpi. Correspondingly, also ChlIdx displayed a significant decrease, while mAriIdx increased considerably from 0 to 4 dpi (**Figure 7B**). The isolates B8, B9, B11, and B14 showed similar temporal changes as the intermediate aggressive isolate B12 for F_v_/F_m_, ChlIdx, and mAriIdx, except for the mAriIdx of B14 (**Supplementary Figure 4**).

The most virulent isolate B13 caused the greatest decrease of F_v_/F_m_, this was already very strong at 3 dpi, while values further decreased to 0.172 at 4 dpi. Simultaneously a clear decrease in ChlIdx and increase in mAriIdx was found (**Figure 7C**). B1, B2, B3, B4, B10, and B15 grouped with B13 based on their virulence on strawberry leaves and they caused the same trends in F_v_/F_m_ and ChlIdx. However, no significant increase was observed in mAriIdx for B1, B3, and B15.

The *Botrytis* isolates resulted in considerable variations in F_v_/F_m_, ChlIdx, and mAriIdx of the inoculated leaves at 4 dpi, this for all the pre-inoculation light quality regimes of the leaves (**Table 2**).

**Table 2:** Effect of inoculation with different *Botrytis* isolates at 4 dpi on F_v_/F_m_, ChlIdx, and mAriIdx in strawberry leaves grown under white, blue, and red LED lights.

Botrytis	White			Blue			Red		
isolates	F_v_/F_m_	ChlIdx	mAriIdx	F_v_/F_m_	ChlIdx	mAriIdx	F_v_/F_m_	ChlIdx	mAriIdx
B1	0.266 ± 0.129^cde^	1.635 ± 0.357^bcde^	1.590 ± 0.234^ab^	0.199 ± 0.188^de^	1.478 ± 0.420^cd^	1.456 ± 0.323^b^	0.342 ± 0.130^d^	22.072 ± 0.389^c^	1.860 ± 0.439 ^ab^
B2	0.318 ± 0.169^bcd^	1.780 ± 0.375^bc^	1.826 ± 0.504^ab^	0.148 ± 0.097^de^	1.383 ± 0.275^cd^	1.628 ± 0.339^ab^	0.376 ± 0.157^cd^	2.130 ± 0.372^bc^	1.761 ± 0.376 ^abc^
B3	0.218 ± 0.066^de^	1.517 ± 0.183^cde^	1.648 ± 0.200^ab^	0.350 ± 0.248^bc^	1.887 ± 0.522^ab^	1.519 ± 0.458^b^	0.498 ± 0.153^bc^	2.263 ± 0.300^bc^	1.435 ± 0.379 ^bc^
B4	0.223 ± 0.116^de^	1.441 ± 0.264^de^	1.567 ± 0.348^ab^	*	*	*	*	*	*
B5	0.275 ± 0.067^cde^	1.647 ± 0.153^abc^	1.820 ± 0.423^ab^	*	*	*	*	*	*
B7	0.637 ± 0.042^a^	2.243 ± 0.194^a^	1.095 ± 0.280^c^	0.599 ± 0.038^a^	2.166 ± 0.209^a^	0.841 ± 0.214^c^	0.712 ± 0.022^a^	2.765 ± 0.275^a^	1.320 ± 0.314^c^
B8	0.373 ± 0.203^bc^	1.719 ± 0.368^bcd^	1.457 ± 0.269 ^abc^	0.377 ± 0.098^b^	1.685 ± 0.218^bc^	1.289 ± 0.220^bc^	0.586 ± 0.074^ab^	2.434 ± 0.254^ab^	1.340 ± 0.229^c^
B9	0.301 ± 0.095^cde^	1.795 ± 0.198^bc^	1.845 ± 0.245 ^a^	0.206 ± 0.082^cde^	1.600 ± 0.135^bcd^	1.603 ± 0.206^ab^	0.502 ± 0.102^bc^	2.390 ± 0.293^bc^	1.365 ± 0.259^c^
B10	0.210 ± 0.109^de^	1.511 ± 0.384^cde^	1.722 ± 0.507 ^ab^	0.192 ± 0.073^de^	1.335 ± 0.222^d^	1.497 ± 0.418^b^	0.559 ± 0.102^b^	2.349 ± 0.368^bc^	1.478 ± 0.394\^abc^
B11	0.444 ± 0.090^b^	1.881 ± 0.187^b^	1.523 ± 0.213 ^abc^	*	*	*	*	*	*
B12	0.263 ± 0.071^cde^	1.686 ± 0.131^bcde^	1.736 ± 0.298 ^ab^	0.095 ± 0.038^e^	1.573 ± 0.161^bcd^	2.017 ± 0.394^a^	0.354 ± 0.071^d^	2.149 ± 0.207^bc^	1.908 ± 0.302^a^
B13	0.173 ± 0.108^e^	1.489 ± 0.178^cde^	1.839 ± 0.425 ^a^	0.192 ± 0.149^de^	1.559 ± 0.338^bcd^	1.668 ± 0.711^ab^	0.387 ± 0.176^cd^	2.078 ± 0.318^bc^	1.736 ± 0.635^abc^
B14	0.375 ± 0.080^bc^	1.806 ± 0.240^bc^	1.440 ± 0.320 ^abc^	*	*	*	*	*	*
B15	0.222 ± 0.109^de^	1.378 ± 0.282^e^	1.400 ± 0.490 ^bc^	0.280 ± 0.069^bcd^	1.545 ± 0.177^cd^	1.605 ± 0.445^ab^	0.544 ± 0.082^b^	2.367 ± 0.211^dbc^	1.491 ± 0.353^abc^

The statistical analysis was conducted by one-way ANOVA with Bonferroni correction (p=0.0036 for white-light-leaves, and p=0.005 for blue- and red-light-leaves) followed by Tukey’s test. Data were collected from approximate 24 leaf discs of 4 replicates at 4 dpi and presented as mean ± S.D. Significant differences between B. cinerea isolates were indicated with different letters. * means that the isolates were not tested on blue- or red-light-leaves.

Leaves inoculated with the nonpathogenic strain B7 (A366) maintained the highest level of F_v_/F_m_ and ChlIdx, while the lowest levels of mAriIdx were observed, this irrespective of the light quality treatments. Leaf inoculation with the virulent B13 resulted in the strongest decrease of F_v_/F_m_ for white-light-leaves, but not for the other light quality pretreatments. Here, F_v_/F_m_ was lowest after B12 inoculation of leaves grown under both blue and red light. Leaf yellowing and chlorophyll content, assessed by ChlIdx was lowest after B15 inoculation of white-light-leaves, B10 inoculation of blue-light-developed leaves, and B1 inoculation of red-light-developed leaves. Increase of anthocyanins (mAriIdx) was highest in both B9 and B13 in white-light-developed leaves, and in B12 in both blue- and red-light leaves (**Table 2**).

Light pretreatment effects are thus clearly present and these effects are shown in **Table 3**. Overall red pretreated leaves have a significant higher F_v_/F_m_ (p < 0.001) and ChlIdx (p < 0.001), while no difference between blue and white pretreated leaves is found. Only for strains B1 and B13 this positive effect of red light to maintain higher F_v_/F_m_ levels was not observed, while this was the case of B2 with respect to ChlIdx. Overall no significant effect of light pretreatment was found for mAriIdx (p = 0.41), indeed only for 3 out of 10 isolates an effect of light pretreatment of the leaves was present, in B1 and B7 mAriIdx increased significantly while for B9 a significant decrease was found.

**Table 3:** Effect of light quality on the virulence of the *B. cinerea* isolates assessed by chlorophyll fluorescence imaging (F_v_/F_m_) and image-based indices (ChlIdx, and mAriIdx) at 4 dpi.

*Botrytis* isolates	F_v_/F_m_			ChlIdx			mAriIdx		
	White	Blue	Red	White	Blue	Red	White	Blue	Red
B1	0.234a	0.385a	0.349a	1.613a	1.053b	1.832a	1.598ab	1.432b	1.818a
B2	0.299ab	0.108b	0.413a	1.786a	1.441a	1.842a	1.816a	1.626a	1.749a
B3	0.215b	0.508a	0.514a	1.510b	1.913a	2.225a	1.633a	1.526a	1.452a
B7	0.639b	0.592c	0.714a	2.327ab	2.230b	2.542a	0.978b	1.019ab	1.261a
B8	0.395b	0.379b	0.577a	1.724b	1.647b	2.376a	1.464a	1.290a	1.342a
B9	0.306b	0.208c	0.498a	1.882b	1.596c	2.264a	1.779a	1.702a	1.374b
B10	0.207b	0.182bc	0.558a	1.541b	1.342b	2.336a	1.686a	1.604a	1.486a
B12	0.235b	0.075c	0.356a	1.716ab	1.581b	1.974a	1.735a	2.045a	1.826a
B13	0.176a	0.204a	0.312a	1.490b	1.572b	2.164a	1.875a	1.663a	1.763a
B15	0.189c	0.306b	0.545a	1.449b	1.545b	2.597a	1.379 a	1.598a	1.486a

The assessment was performed for strawberry leaves that developed respectively under full spectrum white LED light (white), monochromatic blue LED (blue), and monochromatic red LED (red) light. As light quality influences metabolite levels in the leaves, ANCOVA was performed and adjusted means were calculated with Bonferroni as confidence interval adjustment and the covariates are the values of F_v_/F_m_, ChlIdx, and mAriIdx at 0 dpi.

Data are means of 24 replicates. Different letters indicate significant differences for each isolate between the light treatments per parameter.

Additionally, overall comparison of anthocyanins with respect to the different light qualities (**Supplementary Table 2**) or more specifically for three representative *B. cinerea* isolates (**Supplementary Table 3**) were performed from 0 to 4 dpi. Blue light-developed leaves resulted in significant lower basal anthocyanin levels compared to leaves developed under white and red lights (**Supplementary Table 2**). A higher anthocyanin content was observed in *Botrytis* isolates with higher virulence (**Supplementary Table 3**).

**Figure 8:**
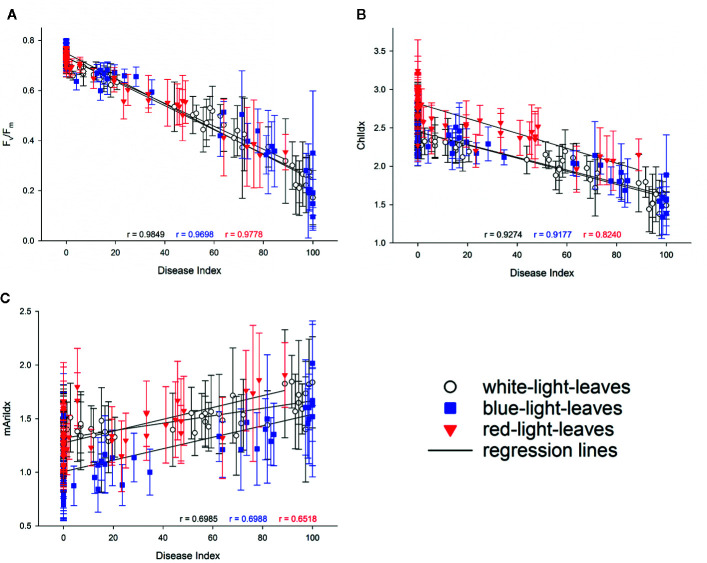
Correlations between disease index and F_v_/F_m_ **(A)**, ChlIdx **(B)**, and mAriIdx **(C)** on strawberry leaves from white (in white), blue (in blue), and red (in red) LED lights. Values are presented as means with standard deviation shown by vertical bar. The Pearson correlation r is shown in colors to indicate the corresponding light treatments.

As virulence of the isolates differed, correlations between the disease index and F_v_/F_m_, ChlIdx, and mAriIdx were explored (**Figure 8**). High correlations between disease index and F_v_/F_m_, were found, this irrespective of the light quality pretreatment (all r ≥ 0.978, **Figure 8A**). Stress indices resulted in lower though still significant correlations between the disease index and ChlIdx although the correlation was lowest for red pretreated leaves (r > 0.917 for leaves from white light and blue light, while r is 0.824 for leaves from red light, **Figure 8B**). Correlations between disease index and mAriIdx were much lower (r < 0.699, **Figure 8C**).

We also checked the correlations between F_v_/F_m_ and phenotypic characteristics of the different isolates such as growth rate (**Figure 1**) and medium acidification (**Figure 4**). Virulence and medium acidification were highly correlated (**Supplementary Figure 7A**), while no correlation between virulence and mycelial growth rate was observed (**Supplementary Figure 7B**).

## Discussion

### Phenotypic Variability of *Botrytis cinerea* Isolates Is Influenced by Light Quality

The 15 *Botrytis* isolates in this study differed in their mycelial growth rate and in their ability to reproduce by conidia or form sclerotia (**Figures 1** **–** **3**, **Supplementary Table 1**). This intraspecies variation in *B. cinerea* is well documented (Martinez et al., 2003; Mirzaei et al., 2009) and can be influenced by environmental factors. As *Botrytis* is an important pathogen in greenhouse plant production, changes in the greenhouse environment might also lead to phenotypic variations. Here, we specifically focused on the increasing application of monochromatic red and blue light and its combination in lighting strategies with LED. Indeed, *B. cinerea*, possesses 11 photoreceptors to sense the surrounding light environment, which triggers various photoresponses influencing vegetative growth, sporulation, germination of spores, and sclerotia formation (Schumacher, 2017). Generally, the short UV A and B (300–420 nm) wavelengths retard the mycelial growth of *B. cinerea* (Tan and Epton, 1973). Here, the shortest wavelength we investigated was monochromatic blue light (460 nm) though this wavelength did not retard the growth rate of the studied isolates, in comparison to the full-spectrum white light. Light quality had a limited effect on the growth rate. Canessa et al. (2013) found that light reduced the daily growth rate of B05.10 (= B6) and attributed this to light-induced stress. It seems that our applied lower light intensities did not induce this light stress except for the strain B1 which showed the highest mycelial growth rate under dark condition. The reduced mycelial growth rate under daylight might be explained by the lower temperature (18°C while 20°C in the climate rooms) as indicated by Tan and Epton (1973).

Early studies showed that sporulation happens exclusively in light, it is strongly stimulated by near-UV light and slightly stimulated by red light, while blue light is ineffective (Tan and Epton, 1973). This light-dependent effect on sporulation was later confirmed for strain B05.10 (= B6) by Canessa et al. (2013) where broad spectrum light (400–720 nm) induced sporulation but blue light inhibited the formation of conidiophores. Also in our research all isolates sporulated under full spectrum daylight (from class I to class V). Monochromatic red light resulted in similar or lower sporulation in most isolates (12 out of the 15), this coincides with the slight stimulation of sporulation by red light reported by Tan and Epton (1973). Sporulation under white LEDs was low, only B1 and B7 sporulated under this light source (both class II) although the light spectrum is very similar to daylight. However, daylight is rich in far-red light (700–800 nm, **Supplementary Figure 1**), which promotes sporulation (Tan, 1975).

Here, the capacity of blue light to inhibit sporulation was only observed in 9 of the 15 isolates. Blue light stimulated sporulation in B6 (B05.10) and two other grape isolates (B2 and B11), in the nonpathogenic strain B7 (A366), and in two lettuce isolates (B9 and B12). The fact that B05.10 (= B6) produced very few spores (class I) under blue light but also hardly sporulated under daylight, might indicate that despite its genetic stability (Canessa et al., 2013) the strain has mutated. This is also supported by the fact that this strain did not form sclerotia in the dark. Adding red light (600–700 nm) to the blue spectrum did not enhance sporulation as only three isolates sporulated under these conditions. Stewart and Long (1987) reported that *Botrytis* strains could also show varying degrees of sporulation in the dark. This is also confirmed in our study where 8 out of 15 isolates sporulated in the dark. Although blind strains that exhibit the same phenotype under light and darkness are found in nature, this was not the case for our field collections. Only B7, the nonpathogenic mutant A366 produced conidia under each light quality as well as under dark condition and, as described by Kunz et al. (2006), was not able to form sclerotia.

Early studies indicated that sclerotia formation for survival exclusively occurs in cultures in constant darkness. Yet, this can be affected by small broad light dosages. Less sclerotia were formed when irradiated for 30 min compared to 15 min, and none were observed when irradiated for more than 60 min. Furthermore, sclerotia formation was found to be promoted by red and infrared light (Tan and Epton, 1973). In this study, blue, blue+red, and white lights inhibited sclerotia formation in the 15 *B. cinerea* isolates. This inhibition is due to the blue light fraction as under red light sclerotia were formed in 12 out of the 15 isolates. In this study also daylight at a very low light fluence (10 µmol m^−2^ s^−1^) induced sclerotia promotion which can be caused by the enrichment of the longer wavelengths (red light or the far-red light) in the indoor daylight spectrum (Tan and Epton, 1973).

It is clear that phenotypic responses of *B. cinerea* isolates to light quality are very diverse and sometimes conflicting with earlier publications. These conflicting results might be due to the fact that limited strains were studied though probably also light intensity might be an interacting factor with light quality. Also day length effects cannot be excluded, most studies apply a photoperiod of 12 h while in this research a photoperiod of 16 h, based on greenhouse lighting duration was applied.

### Virulence Variation in *Botrytis cinerea*

*B. cinerea* isolates in this study displayed significant variations in their virulence. Virulence diversity in *B. cinerea* isolates has been studied in various locations and on various plants around the world (Kerssies et al., 1997; Martinez et al., 2005; Mirzaei et al., 2009; Kumari et al., 2014). Variation in virulence of *B. cinerea* is often due to differences in cell wall degrading enzymatic activities and in the secretion of other virulence factors such as oxalic acid (Derckel et al., 1999). Higher oxalic acid accumulation leads to higher aggressiveness of the pathogen and reversely, lower secretion of oxalic acid is associated with lower virulence (Kunz et al., 2006; Sun et al., 2019). Various degrees of medium acidification caused by oxalic acid production of *B. cinerea* isolates were also observed in this study (**Figure 4**). B4, B13, and B15 with highest virulence on strawberry leaves secreted more oxalic acid. In contrast, B7 (A336), the nonpathogenic mutant, showed no oxalic acid production and did not cause disease. Medium acidification and virulence were highly correlated (**Supplementary Figure 7A**). Y*et al*so other virulence factors such as cell wall degrading enzymes, toxins, and secondary metabolites (Sharma and Kapoor, 2017) can explain differences in virulence. However, it seems that *B. cinerea* isolates with strong virulence favored less sporulation and produced more sclerotia (**Figure 3**).

### Strawberry Leaves Developed Under Red Light Displayed Enhanced Resistance to *Botrytis cinerea* Isolates

Higher resistance to all the tested *B. cinerea* isolates (except for the non-pathogenic B7) was found in strawberry leaves grown under red LEDs (**Figure 6**). Meng et al. (2019) showed that red LED light increased strawberry leaf basal resistance against R16 (B1 in this study). Resistance improvement by red light was associated with lower hydrogen peroxide levels in the red-light-developed leaves of strawberry (Meng et al., 2019). It might also be linked to phytochrome modulated defense signaling where low R:FR ratios repress the jasmonate response to *Botrytis* (Cerrudo et al., 2012; Ballaré, 2014). In contrast blue-light-developed leaves were more susceptible to some of the isolates, including B1, B2, B9, B12, and B13 (**Figure 6**) and had lower level of anthocyanins compared to leaves grown under white and red lights (**Supplementary Table 2**). Here, both the increased hydrogen peroxide level as well as the reduced anthocyanin level might explain the increased susceptibility (Bassolino et al., 2013; Meng et al., 2019).

### F_v_/F_m_ Is the Best Indicator for Early Detection of *Botrytis cinerea* on Strawberry Leaves

Virulence of the *Botrytis* isolates on strawberry leaves derived from different light quality pretreatments was also assessed by imaging. The decrease in F_v_/F_m_ synchronized with the development of lesion size, which was visualized on fluorescence images in dark red (**Figure 7**, **Supplementary Figures 4** and **5**). Strong correlations between F_v_/F_m_ and disease index were observed in strawberry leaves derived from white (r = 0.985), blue (r = 0.97), and red (r = 0.978) LEDs (**Figure 8**).

F_v_/F_m_ responded well to the development of *Botrytis* disease in strawberry leaves. Both biotic and abiotic stress factors decrease the efficiency of photosynthesis and suppress the variable fluorescence of dark-adapted chlorophyll-containing leaves correspondingly. F_v_/F_m_ decreases along with the increasing effect of stresses (Rolfe and Scholes, 2010; Gorbe and Calatayud, 2012). This reduction in F_v_/F_m_ suggests destructive changes in chloroplasts and photosystem II caused by *B. cinerea* infection. Here ChlIdx was clearly not as sensitive as F_v_/F_m_, only at higher disease indices (> 50%) this parameter decreased. Furthermore, F_v_/F_m_ could discriminate virulence of the *B. cinerea* isolates. Higher virulence resulted in a stronger decrease in F_v_/F_m_, and lower virulence in smaller F_v_/F_m_ reduction. In this study, the infectious symptom by *B. cinerea* were predominantly observed at 2 dpi based on chlorophyll fluorescence image. Chaerle et al. (2007) also showed that chlorophyll fluorescence imaging can be used for early detection of *Botrytis* infection. Autofluorescence signals (F_440_/F_740_) after *Botrytis* inoculation on grape berries could only be recorded 4 days after infection (Bélanger et al., 2011), which is a delay of 2 days in comparison with F_v_/F_m_ and 1 day in comparison with ChlIdx response.

Anthocyanins are water-soluble pigments responsible for the red colors in leaves. Different abiotic stresses enhance the biosynthesis of anthocyanins in leaves, and anthocyanins act as antioxidant and reactive oxygen scavengers (Landi et al., 2015). Heim et al. (1983) described that zones of anthocyanin accumulation often surround restricted lesions where a plant disease has been successfully contained, whereas low anthocyanin levels often occur in susceptible combinations of maize. Additionally, anthocyanins levels are negatively correlated with the susceptibility to *B. cinerea* in tomato fruit (Bassolino et al., 2013). *B. cinerea* infection causes hydrogen peroxide accumulation which leads to the accumulation of anthocyanin at the infectious site (Chalker-Scott, 1999; Govrin and Levine, 2000). In this study, foliar anthocyanins are indicated by mAriIdx. Along with the pathogen invasion, anthocyanins accumulated at the infectious site as visualized in **Figure 7** and **Supplementary Figures 4** and **5**. Despite the antioxidative role of anthocyanins responding to stresses, few study focuses on its development along with pathogen infection by imaging technology. Here the blue-light-leaves which had an increased susceptibility to *Botrytis*, showed significant lower basal anthocyanin level compared to leaves derived from white and red lights (**Supplementary Table 2**). On the other hand, *B. cinerea* isolates with higher virulence induced a higher anthocyanin content (**Supplementary Table 3**), this was due to the higher accumulation of hydrogen peroxide caused by *Botrytis* infection. Furthermore, weaker correlations between *Botrytis* disease index and anthocyanin level are observed, compared to F_v_/F_m_ and ChlIdx. Therefore, it seems that AriIdx is more a supporting observation, but less useful for the early detection.

### Horticultural Implications

*B. cinerea* causes significant losses in plant greenhouse production, storage, shipping, and marketing, which makes control of gray mold very important (Michailides and Elmer, 2000; Karchani-Balma et al., 2008; González et al., 2009). To control this pathogen cultural, chemical, and biological methods, as well as plant resistance breeding are used (Pande et al., 2006). However, the complexity and variability of this pathogen are reasons that make control difficult (Mirzaei et al., 2009). Awareness of the existence of pathogen variability together with the new insights in photobiological responses of both pathogen and plants may contribute to more efficient non-chemical methods of control. Here it is confirmed that red LED irradiance improved leaf resistance not only to one *B. cinerea* strain, but to all tested isolates in this study. Therefore, red LEDs have potential to be used in plant production system to control gray mold but this should be further investigated in greenhouse conditions. Moreover, effects of red LED irradiance on *Botrytis* infections on strawberry flowers and fruits need to be assessed.

Many biotic stress symptoms start as spots or patches within a crop. Such symptoms could be discovered with imaging techniques at an early stage, when no visible symptoms are yet apparent. The possibility of early stress detection allows timely treatment to prevent pathogen spread within the crop and greenhouse, which would result in limited yield loss and reduced chemicals usage. Today robots equipped with sensors are under investigation in greenhouse production. A UV-Robot with UV-C radiation is developed to control powdery mildew in horticulture (Mazar et al., 2018). This could also be equipped with imaging sensors, as early detection of disease is beneficial from both economic and environmental perspectives. In this study we evaluated imaging sensors in a highly controlled environment, without interference from other environmental factors such as wind, fluctuating light intensities, and temperature. Moreover, in horticultural production systems, not only environmental factors change in a dynamic way but also crop-dependent factors such as leaf morphology and orientation, leaf waxes and hairs, and leaf density might influence the response. Therefore, the potential of early detection of gray mold by chlorophyll fluorescence imaging in horticulture needs further validation in a greenhouse environment (Gorbe and Calatayud, 2012; Mahlein, 2016).

## Conclusion

Here it is clearly shown that *B. cinerea* isolates differently respond to different light qualities in phenotypes such as mycelial growth, sporulation, and sclerotia formation. Despite differences in virulence, red light considerably improved leaf basal resistance against all the tested *B. cinerea* isolates, while blue light pretreatment increased leaf susceptibility to some of them. Disease development caused by different *B. cinerea* isolates is highly correlated with F_v_/F_m_ (maximal PSII quantum efficiency), meaning that this indicator can be used to objectively quantify *B. cinerea* disease severity and may also be useful for early detection of plant stress related to gray mold infection. Overall, red LED light has potential to control gray mold in greenhouse production, and image sensors could be developed into a new technology for early disease detection.

## Data Availability Statement

The raw data supporting the conclusions of this article will be made available by the authors, without undue reservation.

## Author Contributions

LM, HM, MH, and M-CL conceived and designed the experiments. HM performed the experiments of light quality effects on phenotypic variations in the 15 Botrytis isolates. LM conducted the experiments of pathogenic test of *Botrytis* isolates on strawberry leaves developed under white, blue, and red LED lights and of imaging from the phenotyping platform. MA and KA guided the experiments on the phenotyping platform and image data analysis. LM analyzed the data and drafted the manuscript. HM, MA, KA, MH, and M-CL critically revised the manuscript. LM, MH, and M-CL reviewed and approved the final manuscript.

## Funding

The first author has a grant of the China Scholarship Council (CSC).

## Conflict of Interest

The authors declare that the research was conducted in the absence of any commercial or financial relationships that could be construed as a potential conflict of interest.
