# Supplementary material for: Phenotypic Variation of Botrytis cinerea Isolates Is Influenced by Spectral Light Quality
Source: Front Plant Sci. 2020 Aug 13;11:1233. doi: 10.3389/fpls.2020.01233 (PMC7438557; doi:10.3389/fpls.2020.01233)
Supplement: Supplementary file 1 [file Image_1.pdf]

Supplementary figures

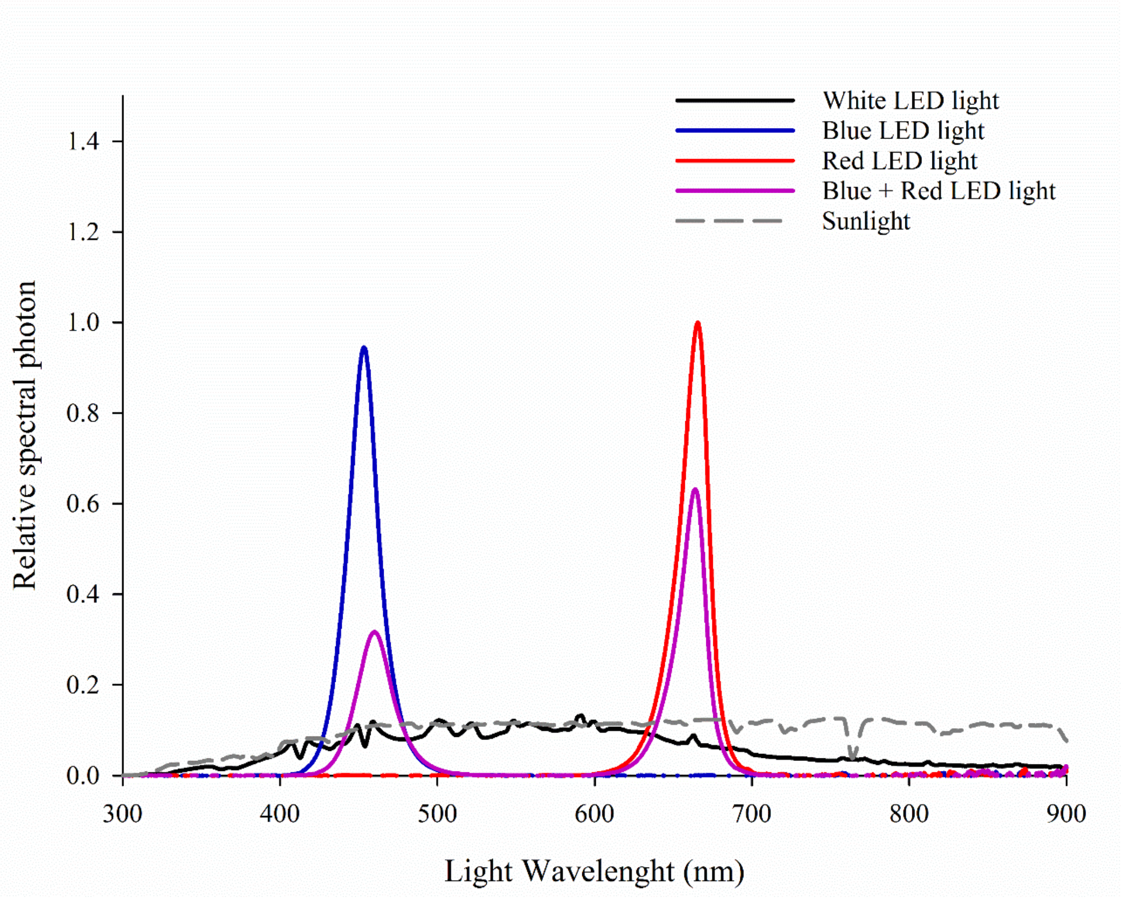

Supplementary Figure 1. Relative spectral photon description of LEDs used for morphology test of B. cinerea isolates and strawberry growth: White LED (white), Blue LED (blue), Red LEDs (red), Red plus blue (red+blue), and sunlight. Spectrum was measured with a JAZ spectrometer (Ocean optic, USA).

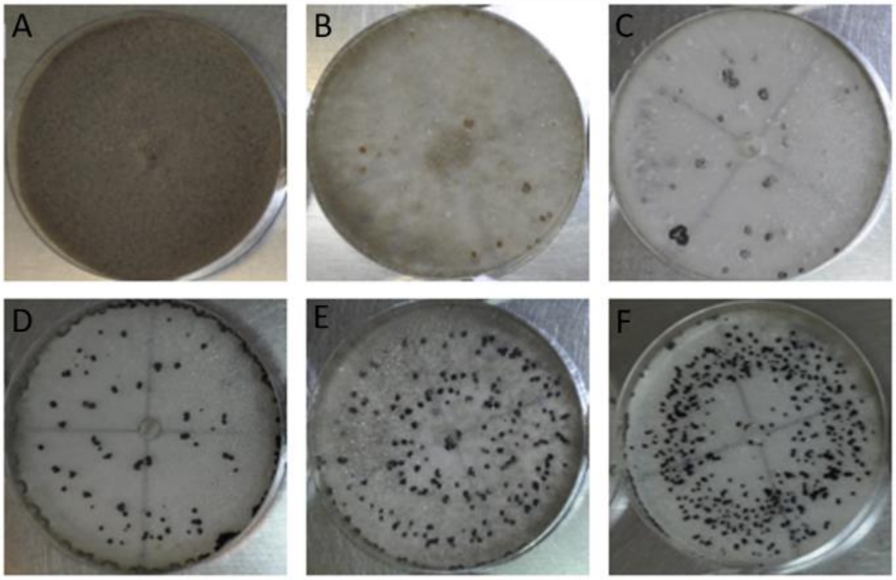

Supplementary Figure 2. Classification of sclerotia production by *B. cinerea* isolates. A: Class 0 (no sclerotia); B: Class I (very few sclerotia); C: Class II (sclerotia formation sparse); D: Class III (average amount of sclerotia); E: Class IV (many sclerotia); F: Class V (abundant sclerotia formation).

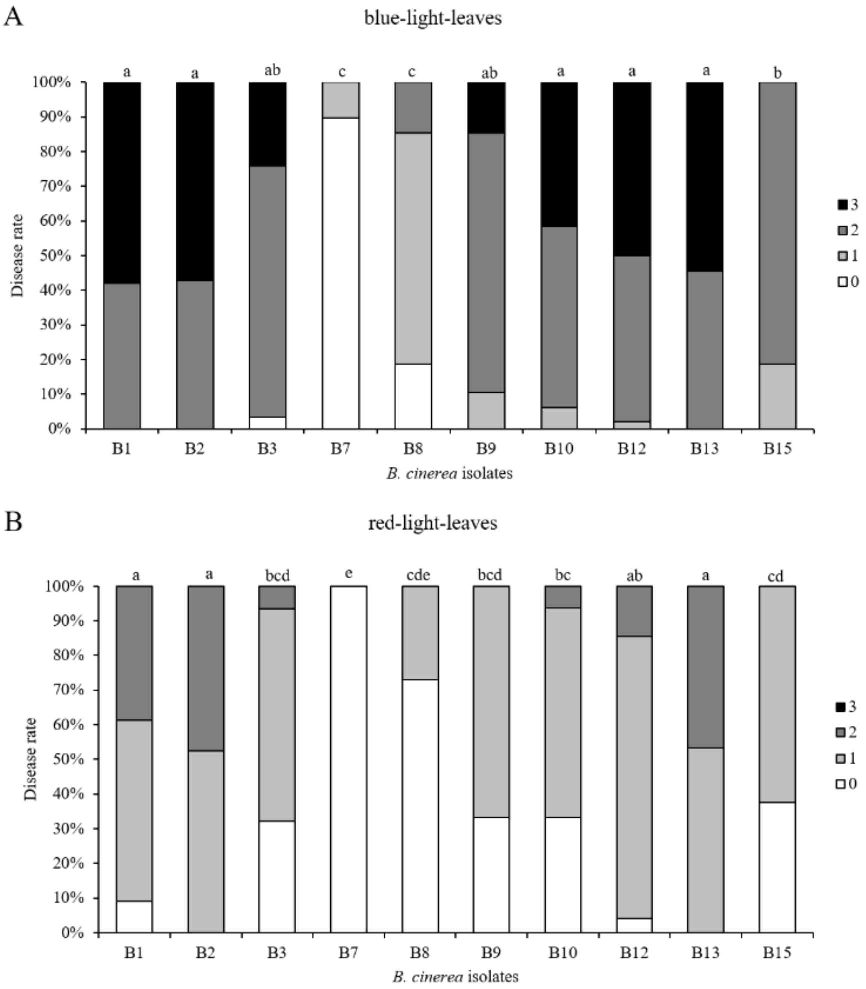

Supplementary Figure 3. Pathogenicity of ten *B. cinerea* isolates on strawberry leaves developed under blue and red LED lights by spore inoculation. Disease rating was scored 3 days post inoculation using four scoring categories (0, resistant; 1, slightly spreading lesion; 2, moderately spreading lesion; 3, severely spreading lesion). Different letters indicate statistical significance among the isolates performed by Kruskall-Wallis followed by a post-hoc Dunn's test with Bonferroni correction ($p \leq 0.005$, $n = 4$).

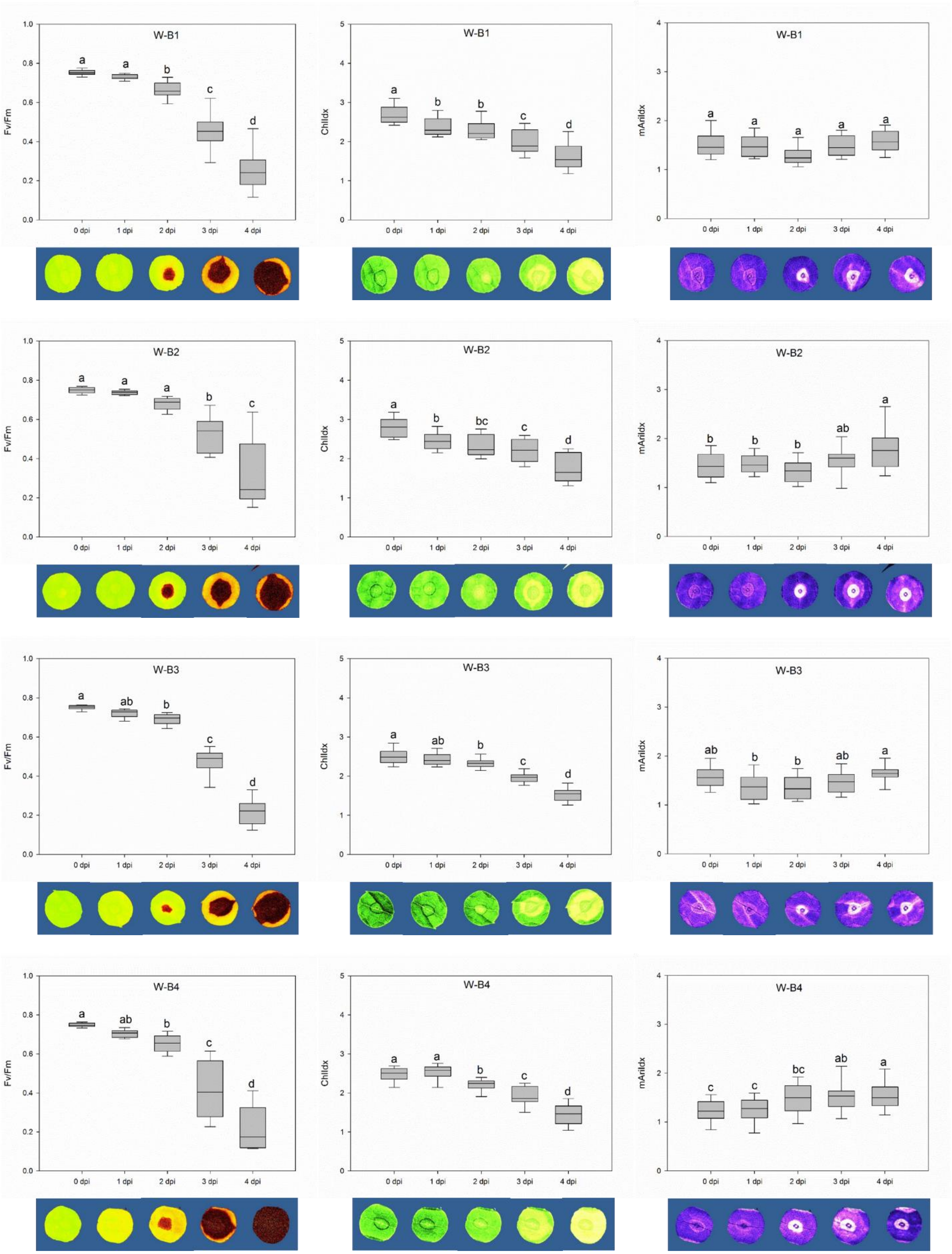

W-B1
Fv/Fm
ChlIdx
mAriIdx
W-B2
W-B3
W-B4
0 dpi
1 dpi
2 dpi
3 dpi
4 dpi

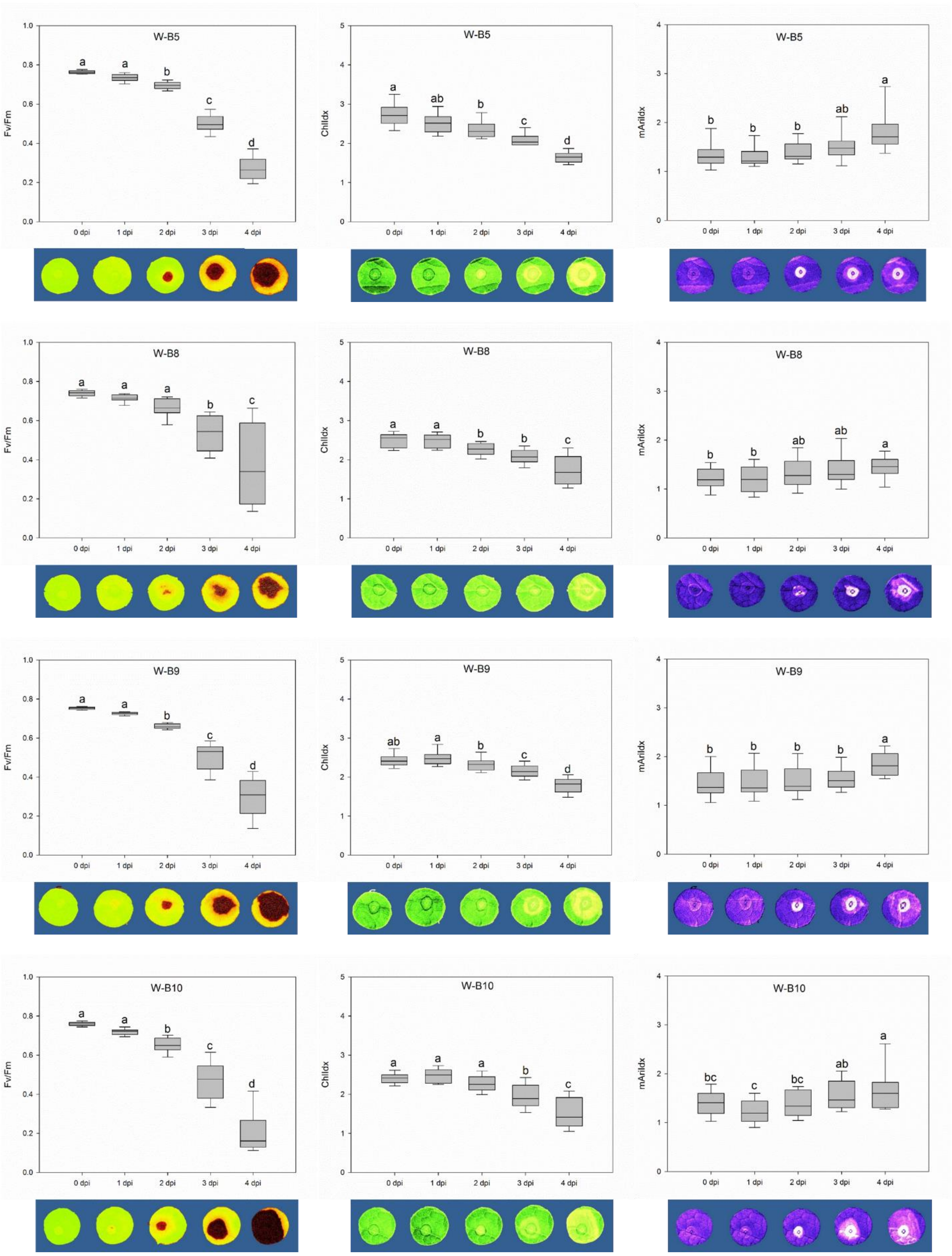
W-B5
W-B8
W-B9
W-B10
Fv/Fm
ChlIdx
mAriIdx
0 dpi
1 dpi
2 dpi
3 dpi
4 dpi

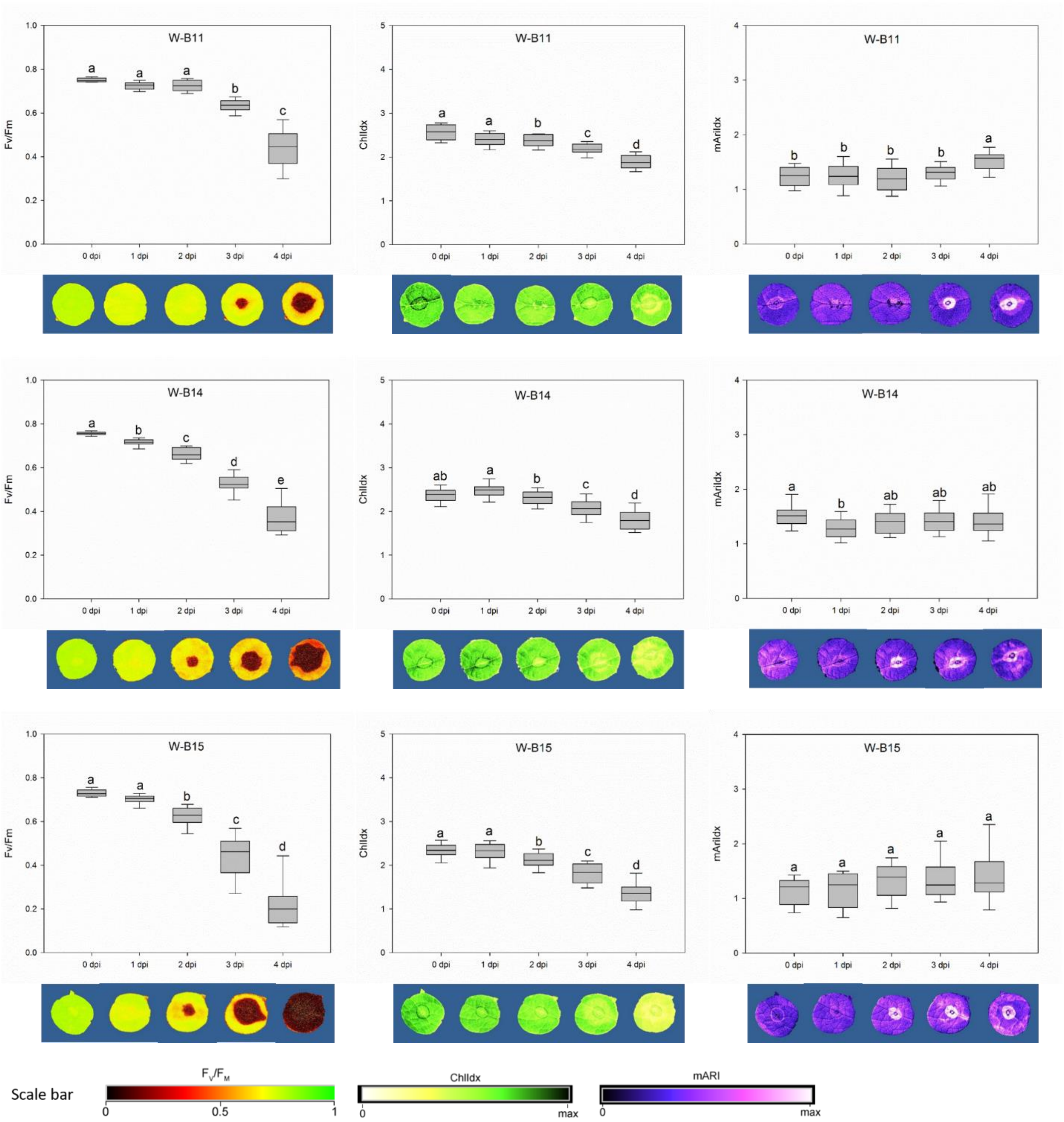

Supplementary Figure 4. The variations of F_v/F_m, ChlIdx, and mAriIdx from 0 to 4 dpi caused by B1, B2, B3, B4, B5, B8, B9, B10, B11, B14, and B15 were correlated with the development of disease lesion on white-light-leaves. The corresponding images are presented underneath the figures. Disease lesion led to darker F_v/F_m image with lower value, yellower ChlIdx image with lower value, and brighter mAriIdx image with higher level. One-way ANOVA was applied for the statistical analysis (Tukey test, $p \leq 0.05$), and data was shown by box plots with median. Different letters indicate significant differences among the time points.

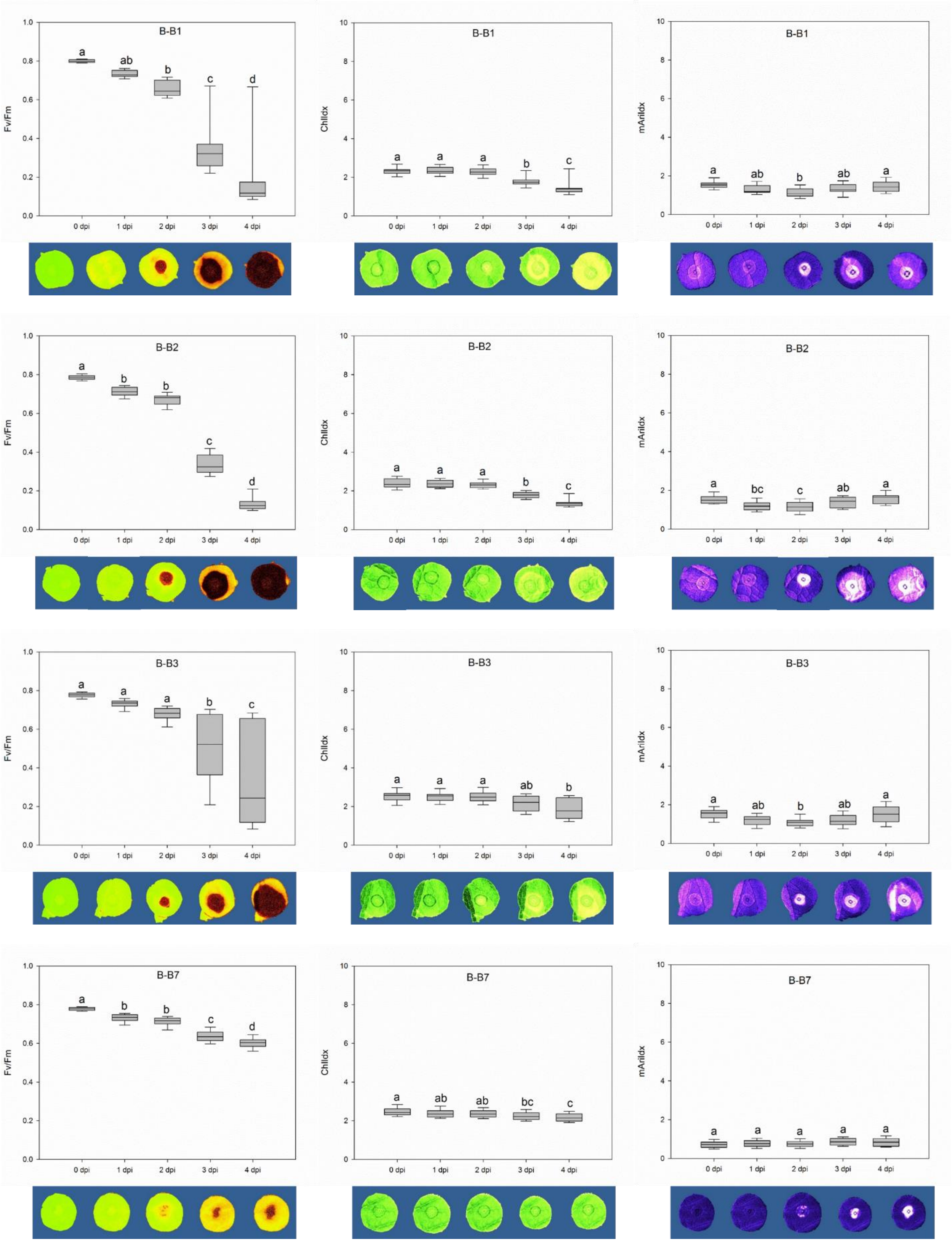
B-B1
Fv/Fm
1.0
0.8
0.6
0.4
0.2
0.0
a
ab
b
c
d
0 dpi
1 dpi
2 dpi
3 dpi
4 dpi
B-B1
ChlIdx
10
8
6
4
2
0
a
a
a
b
c
0 dpi
1 dpi
2 dpi
3 dpi
4 dpi
B-B1
mAriIdx
10
8
6
4
2
0
a
ab
b
ab
a
0 dpi
1 dpi
2 dpi
3 dpi
4 dpi
B-B2
Fv/Fm
a
b
b
c
d
0 dpi
1 dpi
2 dpi
3 dpi
4 dpi
B-B2
ChlIdx
a
a
a
b
c
0 dpi
1 dpi
2 dpi
3 dpi
4 dpi
B-B2
mAriIdx
a
bc
c
ab
a
0 dpi
1 dpi
2 dpi
3 dpi
4 dpi
B-B3
Fv/Fm
a
a
a
b
c
0 dpi
1 dpi
2 dpi
3 dpi
4 dpi
B-B3
ChlIdx
a
a
a
ab
b
0 dpi
1 dpi
2 dpi
3 dpi
4 dpi
B-B3
mAriIdx
a
ab
b
ab
a
0 dpi
1 dpi
2 dpi
3 dpi
4 dpi
B-B7
Fv/Fm
a
b
b
c
d
0 dpi
1 dpi
2 dpi
3 dpi
4 dpi
B-B7
ChlIdx
a
ab
ab
bc
c
0 dpi
1 dpi
2 dpi
3 dpi
4 dpi
B-B7
mAriIdx
a
a
a
a
a
0 dpi
1 dpi
2 dpi
3 dpi
4 dpi

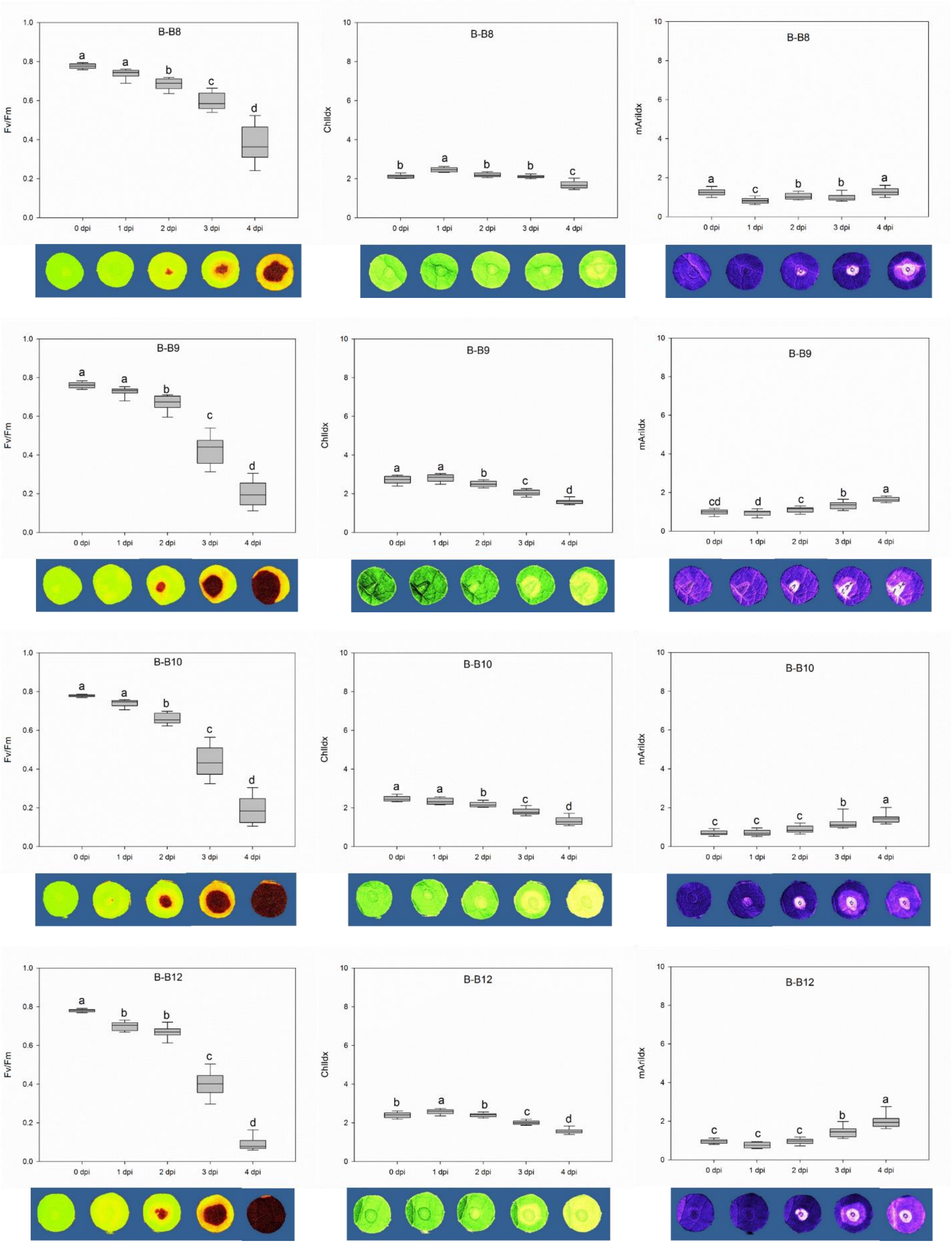

B-B8
B-B9
B-B10
B-B12
Fv/Fm
ChlIdx
mAriIdx
0 dpi
1 dpi
2 dpi
3 dpi
4 dpi

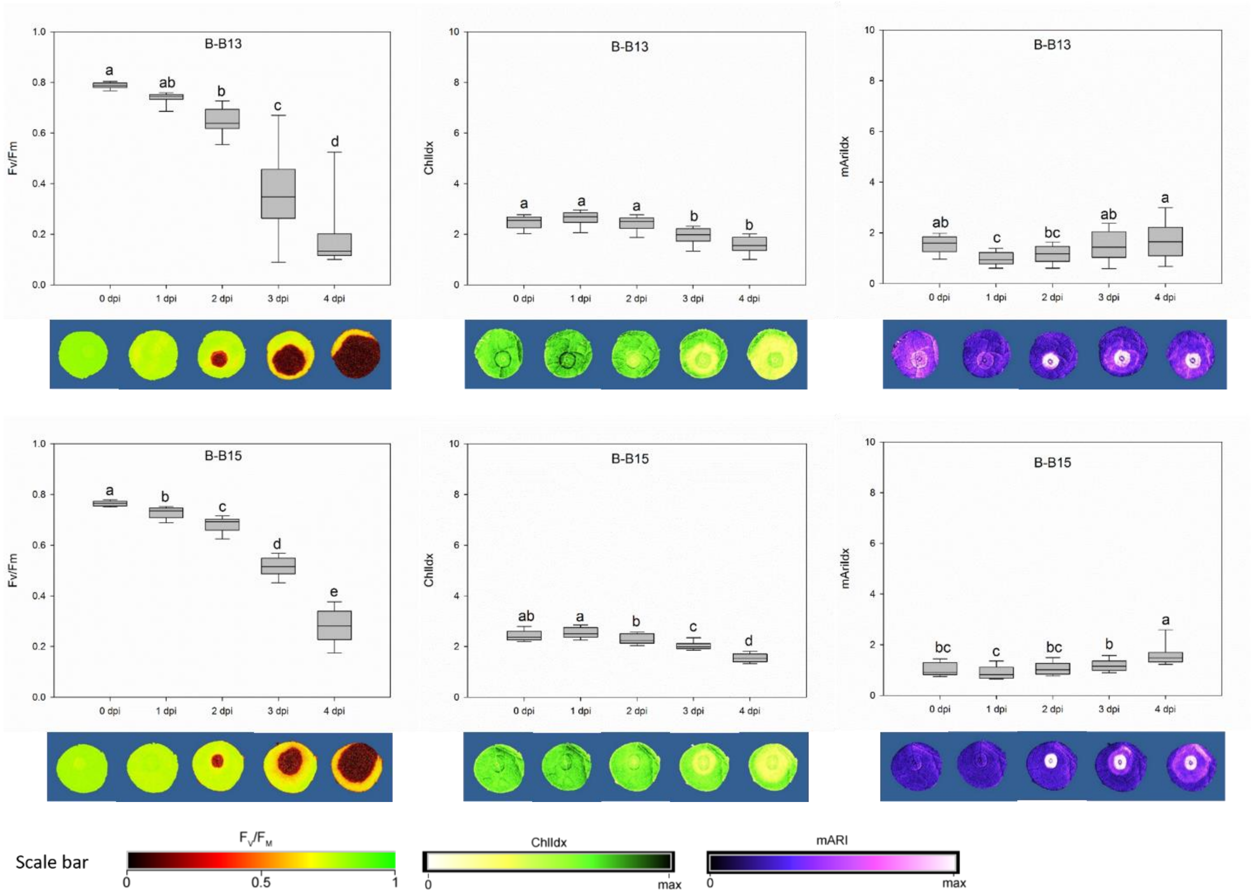

Supplementary Figure 5. The variations of F_v/F_m, ChlIdx, and mAriIdx from 0 to 4 dpi caused by B1, B2, B3, B7, B8, B9, B10, B12, B13 and B15 were correlated with the development of disease lesion on blue-light-leaves. The corresponding images are presented underneath the figures. Disease lesion led to darker F_v/F_m image with lower value, yellower ChlIdx image with lower value, and brighter mAriIdx image with higher level. One-way ANOVA was applied for the statistical analysis (Tukey test, $p \leq 0.05$), and data was shown by box plots with median. Different letters indicate significant differences among the time points.

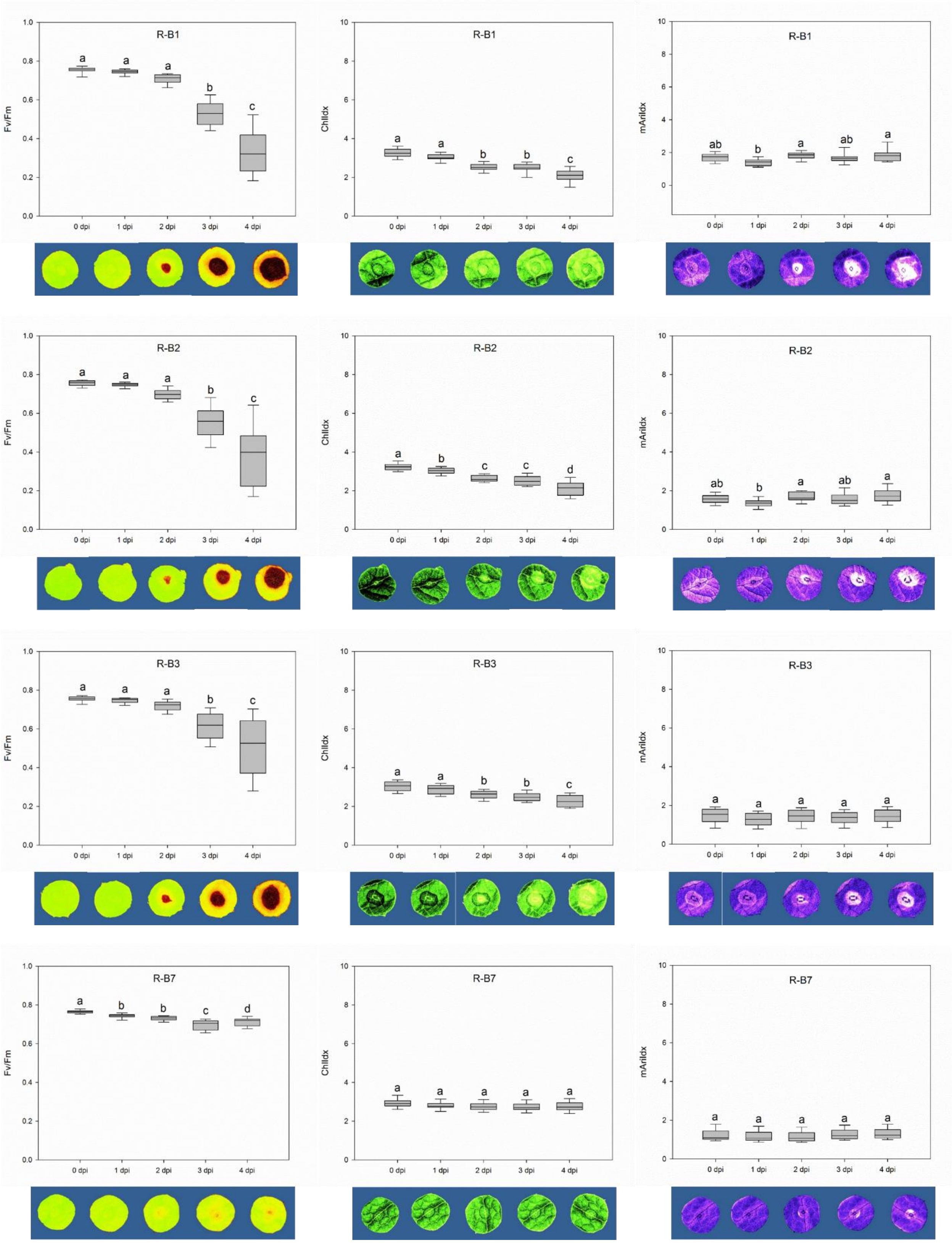
R-B1
R-B2
R-B3
R-B7
Fv/Fm
ChlIdx
mAriIdx
0 dpi
1 dpi
2 dpi
3 dpi
4 dpi
a
a
a
b
c
ab
b
ab
d

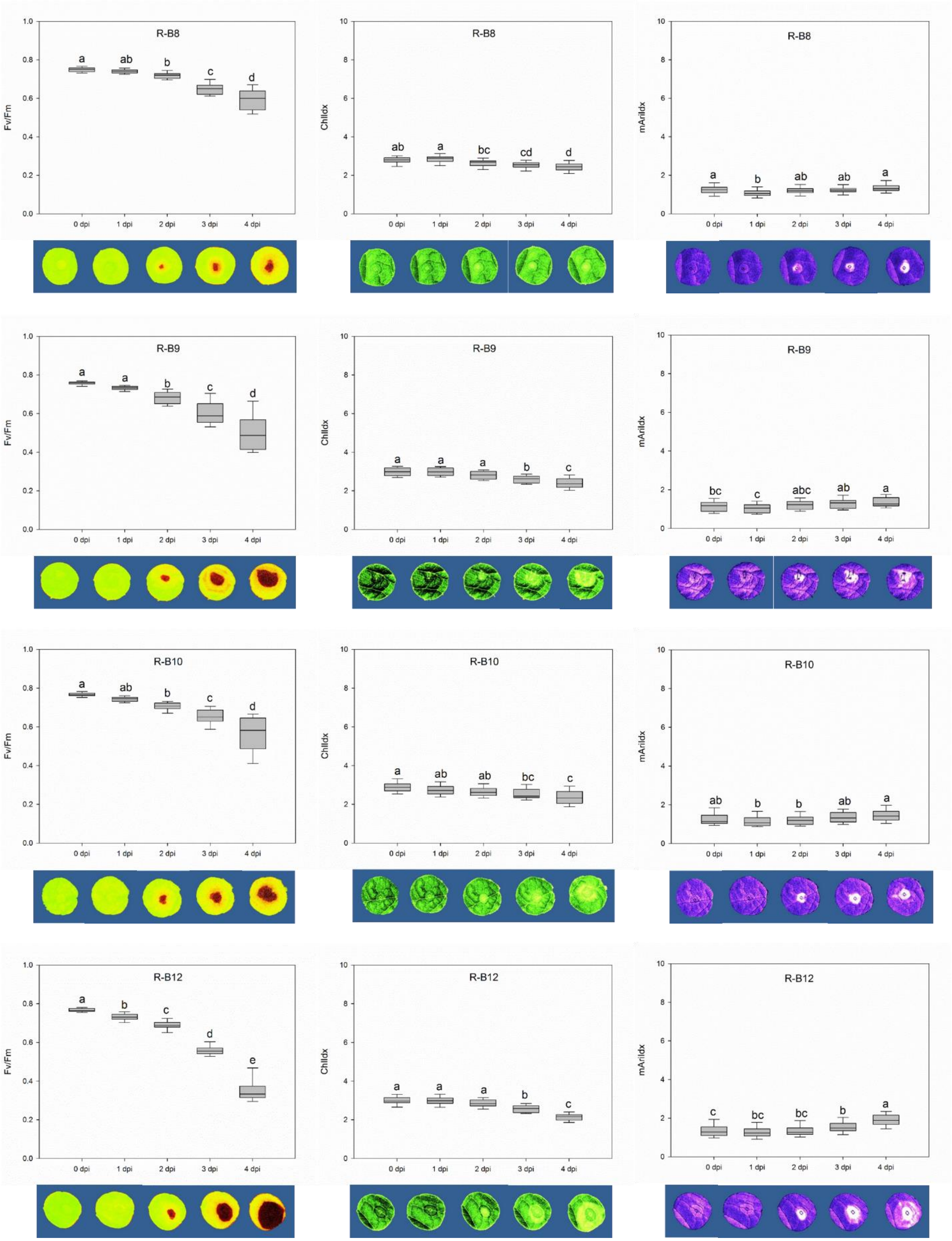
R-B8
Fv/Fm
a
ab
b
c
d
0 dpi
1 dpi
2 dpi
3 dpi
4 dpi
R-B8
ChlIdx
ab
a
bc
cd
d
R-B8
mAriIdx
a
b
ab
ab
a
R-B9
Fv/Fm
a
a
b
c
d
R-B9
ChlIdx
a
a
a
b
c
R-B9
mAriIdx
bc
c
abc
ab
a
R-B10
Fv/Fm
a
ab
b
c
d
R-B10
ChlIdx
a
ab
ab
bc
c
R-B10
mAriIdx
ab
b
b
ab
a
R-B12
Fv/Fm
a
b
c
d
e
R-B12
ChlIdx
a
a
a
b
c
R-B12
mAriIdx
c
bc
bc
b
a

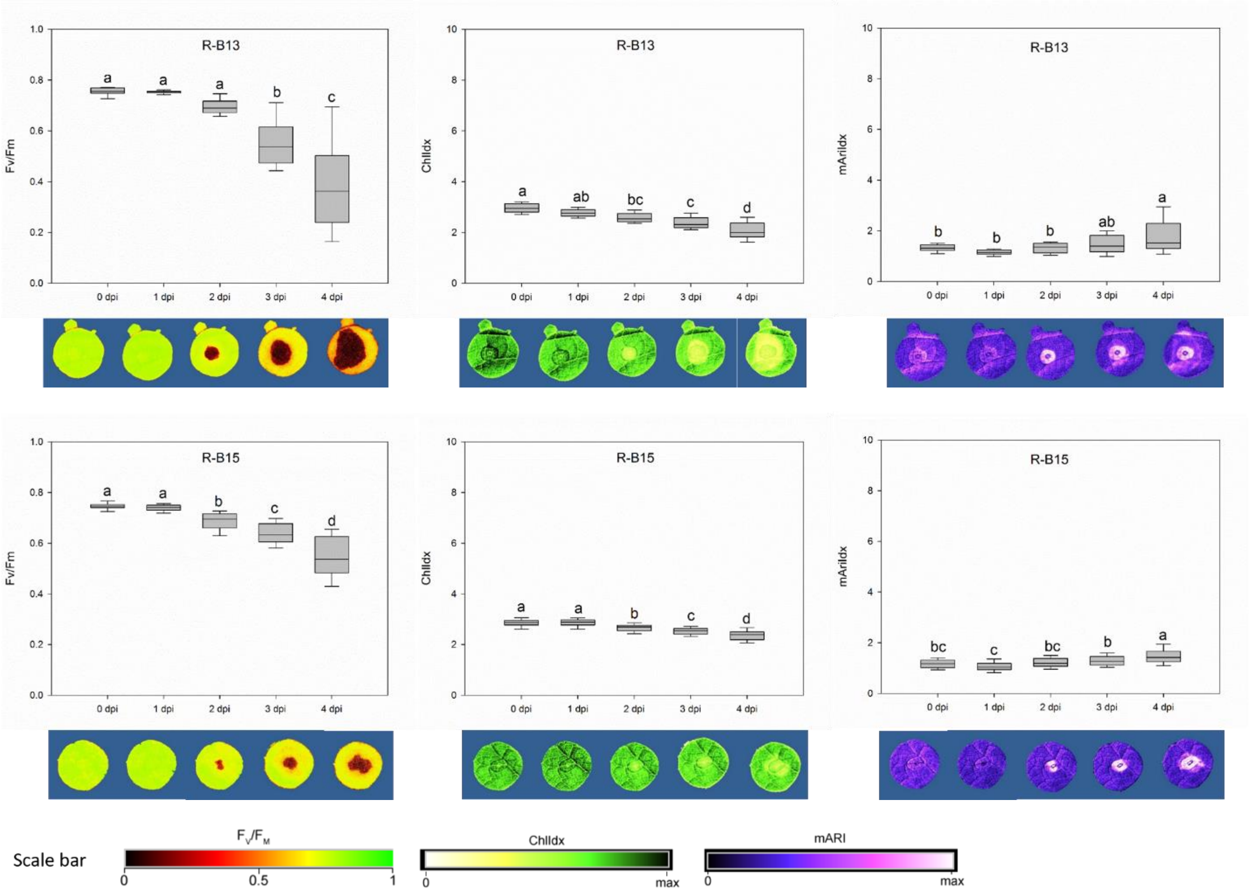

Supplementary Figure 6. The variations of F_v/F_m, ChlIdx, and mAriIdx from 0 to 4 dpi caused by B1, B2, B3, B7, B8, B9, B10, B12, B13 and B15 were correlated with the development of disease lesion on red-light-leaves. The corresponding images are presented underneath the figures. Disease lesion led to darker F_v/F_m image with lower value, yellower ChlIdx image with lower value, and brighter mAriIdx image with higher level. One-way ANOVA was applied for the statistical analysis (Tukey test, $p \leq 0.05$), and data was shown by box plots with median. Different letters indicate significant differences among the time points.

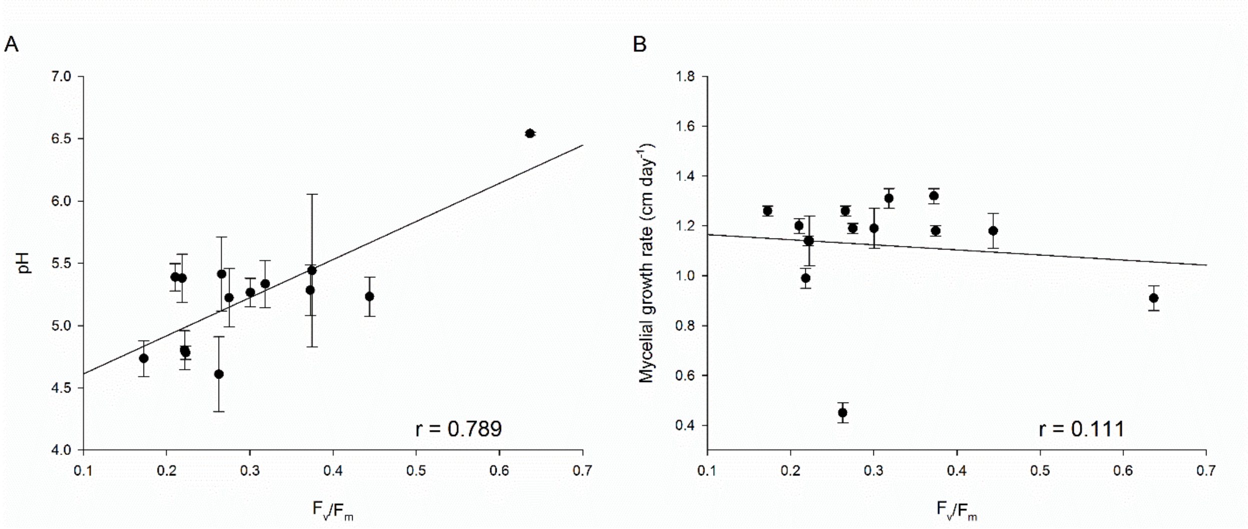

Supplementary Figure 7. The relationship between F_v/F_m and pH (**A**) and mycelial growth rate (**B**). F_v/F_m values observed at 4 dpi from infection in white-light leaves were used for the Pearson correlation (r). Values are presented as means with standard deviation shown by vertical bar.
