# Supplementary material for: Phenotypic Variation of Botrytis cinerea Isolates Is Influenced by Spectral Light Quality
Source: Front Plant Sci. 2020 Aug 13;11:1233. doi: 10.3389/fpls.2020.01233 (PMC7438557; doi:10.3389/fpls.2020.01233)
Supplement: Supplementary file 2 [file Table_1.pdf]

Supplementary Tables:

Supplementary Table 1. Mycelial growth rate of *B. cinerea* isolates under different light conditions.

B. cinerea Isolates	Growth Rate (cm day^{-1})											
	Dark		Daylight		White		Blue		Red		Blue+Red	
B1	1.26±0.02	ab	0.94±0.08	ef	0.86±0.10	ef	0.90±0.12	f	0.94±0.10	e	1.07±0.03	gh
B2	1.31±0.04	a	1.18±0.02	c	1.37±0.03	ab	1.37±0.03	a	1.40±0.09	ab	1.38±0.03	a
B3	0.99±0.04	efg	0.82±0.05	fg	1.29±0.05	cd	1.29±0.02	cd	1.29±0.04	cd	1.21±0.04	fgh
B4	1.14±0.10	def	1.06±0.05	d	1.32±0.08	bc	1.38±0.06	a	1.28±0.04	cd	1.32±0.03	abcd
B5	1.19±0.02	cd	1.08±0.02	d	1.30±0.03	cd	1.27±0.08	cde	1.28±0.04	cd	1.31±0.02	cde
B6	1.24±0.02	abc	1.10±0.03	d	1.32±0.08	bc	1.23±0.10	de	1.40±0.09	ab	1.32±0.03	abcd
B7	0.91±0.05	fg	0.84±0.10	fg	1.22±0.05	def	1.21±0.02	def	1.29±0.05	cd	1.28±0.04	cdef
B8	1.32±0.03	a	1.42±0.03	a	1.37±0.08	ab	1.57±0.06	a	1.37±0.03	ab	1.32±0.03	abc
B9	1.19±0.08	bcd	1.24±0.02	ab	1.47±0.08	a	1.43±0.14	a	1.47±0.03	a	1.35±0.10	a
B10	1.20±0.03	bcd	1.19±0.02	bc	1.32±0.03	bc	1.32±0.03	c	1.23±0.06	cd	1.33±0.06	a
B11	1.18±0.07	cd	1.20±0.00	abc	1.37±0.03	ab	1.42±0.10	a	1.45±0.13	a	1.20±0.13	efg
B12	0.45±0.04	g	0.49±0.02	g	0.59±0.03	f	0.59±0.07	f	0.62±0.08	e	0.66±0.09	h
B13	1.26±0.02	ab	1.19±0.05	c	1.28±0.05	cd	1.26±0.04	de	1.32±0.02	bc	1.26±0.04	def
B14	1.18±0.02	de	1.07±0.03	de	1.27±0.03	cde	1.28±0.02	cde	1.31±0.02	bc	1.28±0.02	cdef
B15	1.14±0.02	de	1.09±0.02	d	1.26±0.02	cde	1.22±002	ef	1.22±0.02	de	1.14±1.13	fgh

Values are means of six replicates ± standard deviation; different letters indicate significant differences based on Kruskal-Wallis test with Bonferroni correction ($P \leq 0.0033$).

Supplementary Table 2. Overall anthocyanin content at 0, 1, 2, 3, 4 dpi of leaves pretreated with different light qualities: white, blue, and red.

Light treatments	0 dpi	1 dpi	2 dpi	3 dpi	4 dpi
white	1.356 a	1.266 a	1.327 a	1.459 a	1.580 a
blue	1.129 b	0.960 c	1.072 b	1.271 b	1.560 a
red	1.343 a	1.172 b	1.335 a	1.389 a	1.552 a

As light quality influences metabolite levels in the leaves, ANCOVA was performed and the covariates are the values of AriIdx at 0 dpi. Different letters indicate significant differences for overall isolates between the light treatments (One-way ANOVA, Tukey's test, $p \leq 0.05$).

Supplementary Table 3. Comparisons of anthocyanin content between representative *Botrytis* isolates: B7 (non-pathogenic strain), B12 (intermediate virulence), and B13 (highest virulence) for white-light-leaves, B7 (non-pathogenic strain), B15 (intermediate virulence), and B2 (highest virulence) for blue- and red-light leaves.

strains	white				strains	blue				red			
	1 dpi	2 dpi	3 dpi	4 dpi		1 dpi	2 dpi	3 dpi	4 dpi	1 dpi	2 dpi	3 dpi	4 dpi
**B7**	1.217a	1.310a	1.305b	1.049b	**B7**	0.787b	0.738b	0.844b	0.871b	1.217a	1.179b	1.296b	1.358b
**B12**	1.026a	1.340a	1.434b	1.769a	**B15**	0.908b	1.067a	1.209a	1.607a	1.109a	1.258b	1.326ab	1.558ab
**B13**	1.275a	1.473a	1.743a	1.852a	**B2**	1.180a	1.151a	1.445a	1.585a	1.287a	1.605a	1.523a	1.657a

Data are means of 24 replicates. To limit the influence of anthocyanin content in different individual leaves, ANCOVA was performed and the covariates are the values of mAriIdx at 0 dpi. Different letters indicates significant differences for each day (One-way ANOVA, Tukey's test, $p \leq 0.05$).
